# Investigation into the efficacy of a commercially available inactivated avian influenza virus (AIV) vaccine (H9N2) through experimental and field assessments, with an emphasis on its compatibility with recent AIV (H9N2) G1-sublineage isolates

**DOI:** 10.1007/s11259-025-10919-1

**Published:** 2025-10-31

**Authors:** Tamer Mahmoud Abdullatif, Ola Hassanin, Walaa Mohamed, Manar AbdelMageed, Ahmed Al-baqir

**Affiliations:** 1 Faculty of Veterinary Medicine, Department of Avian and Rabbit Medicine, Zagazig University, Zagazig, Al-sharqyia, 44511 Egypt; 2 Faculty of Veterinary Medicine, Veterinary Medicine Hospital, Zagazig University, Zagazig, Al-sharqyia, 44511 Egypt; 3 Faculty of Veterinary Medicine, Department of Pathology, Zagazig University, Zagazig, Al-sharqyia, 44511 Egypt; 4 Faculty of Veterinary Medicine, Department of Pathology and Clinical pathology, Badr University in Cairo (BUC), Badr City, Cairo, 11829 Egypt

**Keywords:** LPAI, H9N2, Dose, Oil inactivated, Vaccine, Shedding

## Abstract

Avian influenza H9N2 virus has established itself as endemic in various parts of the globe notably in the Middle East with significant economic repercussions. Vaccination with inactivated vaccine is regarded as one of the most effective strategies for disease prevention. To enhance the efficacy of the vaccination protocol the seed strain must possess genetic and antigenic characteristics closely resemble to the circulating field viruses. This research sought to assess the effectiveness of an oil-inactivated avian influenza virus (AIV) H9N2 vaccine given at different dosages and ages in broiler chicks as well as in commercial layer chicken flocks. This assessment involved challenging the chicks with the H9N2 G1 lineage avian influenza virus (A/chicken/Egypt/v1896/2022 (H9N2)) at 14 and 21 days of age alongside conducting serological monitoring in a layer flock. Phylogenetic analysis of the vaccine seed strains revealed to more than 99.00% amino acid identity to the recently isolated strains. All applied vaccination protocols successfully reduced virus shedding, clinical symptoms, and pathological damages in the trachea and lung. Birds that received a full dose of the inactivated H9N2 vaccine at zero and three days of age exhibited the strongest immune responses by 28 days even in the presence of maternal-derived antibodies. Furthermore, administering the inactivated H9N2 vaccine at three days of age provided the most substantial clinical protection against challenges occurring at various time points. Implementing a priming-boost vaccination strategy for layers provided the birds with adequate humoral immune responses to achieve sufficient pre-laying titres.

## Introduction

The H9N2 avian influenza virus (AIV) was first isolated in 1966 from turkey flocks in Wisconsin, United States (Homme et al. 1970). Since then, this virus has been detected in various avian species as well as in mammals (Li et al. 2017; Hu et al. 2019; Chen et al. 2024). Phylogenetically, the virus is divided into two primary lineages: Eurasian and American (Xu et al. 2007). The American lineage of H9N2 AIVs predominantly occurs in wild birds, while the Eurasian lineage of H9N2 is further subdivided into multiple genetic sublineages. Notable among these are the G1 sublineage, exemplified by the A/quail/Hong Kong/G1/1997; the Y280 sublineage, represented by A/duck/Hong Kong/Y280/1997; and the Korean Y439 sublineage, indicated by A/duck/Hong Kong/Y439/1997, all of which are regarded as parental H9N2 AIV viruses (Lee and Song 2013).

Avian influenza viruses (H9N2) subtype belonging to the G1 sublineage are the most prevalent strains identified in domestic poultry, particularly in Middle Eastern countries such as Egypt, Saudi Arabia, the United Arab Emirates, and Jordan (Aamir et al. 2007). The first identification of AIV (H9N2) in Egypt took place in 2011 at a bobwhite quail farm (quail/V3413/2011) (El-Zoghby et al. 2012). Over the past 14 years, these viruses have consistently affected poultry farms in Egypt, causing significant economic losses to the poultry industry (Adel et al. 2017; Peacock et al. 2019; Li et al. 2020; Nagar et al. 2023). Egyptian H9N2 viruses are classified into three distinct genotypes. Genotype I was identified in Egyptian poultry populations from 2010 to 2013. In 2014, genotype II emerged due to reassortment between AIV H9N2 G1 and Eurasian strains of AIV H9N2 originating from wild birds. More recently, another reassortment event occurred between genotype II and genotype I viruses, resulting in the classification of a new genotype, designated as genotype III (El Sayes et al. 2022).

On contrary to the pantropic affinity of H5N1, avian influenza virus (H9N2) strains primarily replicate in the respiratory tract. The virus was detected in oropharyngeal swabs from infected and contact-exposed birds between 1 and 7 days post-infection (dpi), followed by regression and eventual absence (Song et al. 2019). This finding indicates the virus’s affinity for the respiratory epithelium and its limited ability to cause systemic disease. Despite this, avian influenza (H9N2) viruses continue to pose significant challenges to the poultry industry, causing substantial economic losses. Several factors can exacerbate infections, potentially leading to systemic disease. These include ongoing viral evolution; coinfection with other pathogens such as infectious bronchitis virus (IBV), Newcastle disease virus (NDV), and Escherichia coli (Naguib et al. 2017); the presence of immunosuppressive diseases like infectious bursal disease virus (IBDV) and Marek’s disease virus (MDV); interference from maternally derived antibodies; and improper vaccination practices. All of these factors can also exacerbate AIV (H9N2) infection (Lu et al. 2001; Li et al. 2003; Capua And Alexander 2008).

A variety of strategies aimed at preventing and managing avian influenza are employed globally, including robust biosecurity protocols and the culling of infected bird populations (Kye et al. 2021; Alqazlan et al. 2022). Nonetheless, the implementation of rigorous vaccination protocols is crucial for effective disease control at the international level.

Several types of vaccines have been utilized, including inactivated vaccines (Suarez And Pantin-Jackwood 2017; Astill et al. 2018), reverse genetics vaccines (An et al. 2019) and recombinant virus vector vaccines (St Paul et al. 2011). Despite these alternatives, oil-based inactivated vaccines remain the most effective method for disease prevention since the virus’s emergence. For a vaccine to achieve its intended efficacy, the seed strain must possess genetic and antigenic characteristics closely aligned with those of currently circulating viruses (Choi et al. 2008). A potential mismatch between circulating strains and existing vaccines results in partial protection and the silent circulation of viruses, thereby increasing the risk of the emergence of new variants. For example, the inactivated oil-adjuvant AIV (H9N2) vaccine was approved in Korea alongside several new technology vaccines. However, it has been reported that the Korean AI (H9N2) virus underwent antigenic drift, evolving into distinct antigenic variants capable of evading vaccine-induced protection (Lee and Song 2013; Islam et al. 2025). A similar situation exists in Egypt, where despite the intensive use of various vaccines and vaccination strategies in poultry farms, AIV continues to evolve through ongoing genetic drift (Nagar et al. 2023). Sequencing analysis of AIV H9N2 isolated from chickens in Egypt in 2021 revealed two mutations in antigenic sites A and B of the HA protein, compared to the parental Egyptian virus (Bedair et al. 2024).Therefore, improved vaccination strategies, including periodic updates of vaccine seed strains, are necessary to achieve effective control and eventual eradication of AIV (H9N2). Furthermore, vaccination should be part of a comprehensive integrated approach to disease control, incorporating continuous nationwide surveillance and farm biosecurity strategies. Another important consideration is that selected strains should demonstrate strong immunogenic properties and exhibit high growth capacity in specific pathogen-free (SPF) embryonated chicken eggs (ECE), enabling the production of large quantities of hemagglutinin (HA) units.

Several earlier experimental studies have indicated that inactivated avian influenza vaccines can induce innate immunity followed by humoral antibody responses. This process offers protection to birds against diseases and mortality caused by avian influenza, as well as mitigating declines in egg production (Capua And Alexander 2008; Raheel et al. 2024). However, the timing of vaccination in meat-type chickens remains a contentious issue, primarily due to their brief lifespan and the influence of maternal immunity levels (Pan et al. 2022). Conversely, for longer-lived birds, such as layer hens and broiler breeders, a two-dose vaccination regimen is deemed appropriate, as it enhances the bird immune responses.

In the present work, a vaccine formulated with inactivated oil-based AIV (H9N2) has been developed and brought to market, utilizing an AIV (H9N2) strain that is phylogenetically aligned with the currently circulating H9N2 viruses. The research examined the vaccine platform, its relationship to field strains, and the seroconversion rates in broiler and layer chickens, both with and without maternal-derived antibodies (MDA). Additionally, the investigation assessed the vaccine’s protective effects against pathological damage, mortality, and virus transmission following a challenge with AIV (H9N2).

## Materials and methods

### Ethical statement

The animal experiment was carried out according to the guidance and regulation of the Zagazig University Institutional Animal Care and Use Committee (ZU-IACUC) with approval number (ZU-IACUC/2/F/94/2024). All the procedures were performed under high biosafety conditions that comply with local animal welfare regulation on experiments with chickens and similar species.

## Experimental birds

Two hundred and fifty six, Cobb-500, broiler commercial chicks were used for the immunogenicity and the two challenges studies. These chicks were housed in a floor-based system within the experimental animal facility at the Faculty of Veterinary Medicine, Zagazig University. Throughout the duration of the experiment, the chicks were provided with unrestricted access to both feed and water. Regular monitoring was conducted to detect any changes that might suggest the onset of infections.

### Vaccines

####  AIV (H9N2) vaccine

- An inactivated AIV vaccine (Premvac Flu H9), which is prepared from inactivation of 9–10 Log_2_/dose of (A/chicken/Egypt//FAO-S33/2021 (H9N2)) strain of G1 sublineage. The administration of the vaccine was conducted through subcutaneous injection in the mid-region of the bird’s neck. Throughout the study, the vaccine was referred to as the PF vaccine.

#### Other vaccines

- A live attenuated (Nobilis Ma5 + ND Clone 30, MSD, Boximeer, The Netherlands\Holanda). It was prepared from infectious bronchitis serotype Massachusetts (strain Ma5): 3.0 log_10_ EID_50_ and NDV vaccine Clone 30: 6.0 log_10_ EID_50_. The vaccine was applied by eye instillation method at zero-day-old chick. The vaccine dose was verified by back titration of the inoculated vaccine.
- A Live IBD virus strain D78 (Nobilis^®^ Gumboro D78 (Intervet, Boximeer, The Netherlands \Holanda) which contain Live IBD virus strain D78: ≥ 4.0 log_10_ TCID_50_.
- Avian influenza and newcastle disease vaccine (HPAI) is produced by HARVAC Biological Technology Company Nanjing, China. The vaccine is prepared from H5N1 subtype (Re-5) vaccine strain and administered subcutaneously at a dose of 0.3 ml/bird to 7 days old chicks.
- Newcastle Disease (ND) Vaccine is manufactured by Merck Sharp & Dohme incorporation, Salamanca, Spain. The vaccine is prepared from a clone selected LaSota strain, B1 type Newcastle disease virus and administered by eye drop route to 17 days old chicks.

### Virus

Avian influenza virus H9N2 G1 lineage (A/chicken/Egypt/v1896/2022 (H9N2)) with an accession number of (OP967482) was used as challenge virus. The infective dose was adjusted to contain 10^6^ embryo infective dose 50 (EID_50_)/0.1 ml and the birds were challenged via the ocular route.

### Animal experiment

#### Study 1: Serological assessment of vaccinated broiler chickens received varying doses of the inactivated AIV vaccine based on strain A/chicken/Egypt//FAO-S33/2021 (H9N2)

A total of one hundred Cobb-500 broiler chicks were allocated into five groups, each consisting of 20 chicks, as detailed in Table 1. Birds from four groups were vaccinated with different doses of the inactivated AIV vaccine A/chicken/Egypt//FAO-S33/2021 (H9N2) via s/c at the bird neck. The four groups of vaccinated birds, designated as A, B, C, and D, received vaccinations of 0.3 cc per bird at zero day of age, 0.3 cc per bird at three days of age, 0.5 cc per bird at three days of age, and 0.5 cc per bird at seven days of age, respectively. A fifth group served as the control and was sham-vaccinated and designated as E/F. Blood samples were obtained weekly from the wing veins, starting at zero day old and continuing until the birds reached 35 days of age.

**Table 1:**
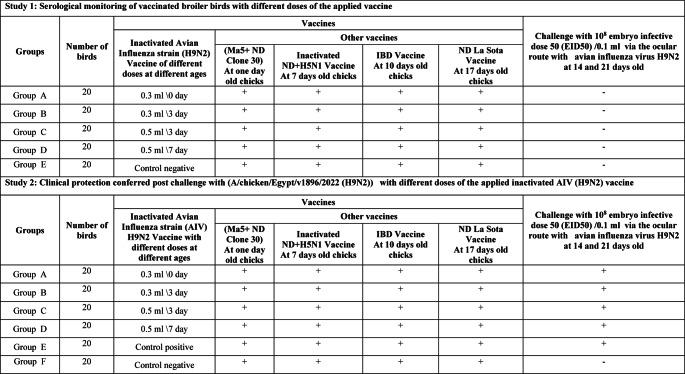
Experimental design

#### Study 2: Clinical protection of vaccinated broiler chickens received varying doses of the inactivated AIV vaccine based on strain A/chicken/Egypt//FAO-S33/2021 (H9N2) conferred post challenge with (A/chicken/Egypt/v1896/2022 (H9N2))

A total of one hundred and twenty Cobb-500 broiler chicks were allocated into six groups, each consisting of twenty chicks (*n* = 20), as detailed in Table 1. Four of these groups were administered varying doses of the Premvac Flu H9 vaccine at different ages. The fifth and sixth groups served as control groups, with no H9N2 vaccination, and were subjected to either a challenge or no challenge. All of the birds were vaccinated against Newcastle disease virus, infectious bursal disease virus, AI (H5N1) virus and infectious bronchitis virus at the defined time and by the appropriate route of vaccination as shown in (Table 1). Birds were subdivided at 14 days old to be challenged with 10^6^ embryo infective dose 50 (EID_50_)/0.1 ml per birds of Avian influenza virus H9N2 G1 lineages (A/chicken/Egypt/v1896/2022 (H9N2)) via the ocular route at either 14 or 21 days old. The birds were monitored daily for a period of 10 days to assess clinical signs of illness and mortality, with all findings meticulously documented. Tracheal swabs were taken on the 3rd, 7th and 10th days post challenge and were preserved at −80 °C for subsequent analysis. Blood samples were drawn from the wing vein (*n* = 10 per group) one week after each challenge, with serum separated and stored at −20 °C. Additionally, three birds from each group were euthanized one week post-challenge, and lung and trachea tissues were collected for histopathological examination. To investigate the transmissibility of the excreted virus in the context of bird immunization, three additional non-immunized birds were introduced as contacts three days after the infection of each group.

### Avian influenza virus H9N2 G1 lineages (A/chicken/Egypt/v1896/2022 (H9N2)) quantification in the tracheal swabs 

Tracheal swabs were obtained (*n* = 6 per group) on the 3rd, 7th, and 10th days following the challenge from labelled vaccinated birds. The swabs were placed in DMEM media and subsequently stored at −80 °C. Viral RNA extraction from the swabs was conducted following the protocol provided by the ABT viral RNA extraction kit (Applied Biotechnology), catalogue number ABT002. Quantitative real-time RT-PCR was performed using the wizpure™ qRT-PCR one-step RT-PCR kit, catalogue number FQP-806-02-78, with specifically designed primer pairs and a probe that targets the HA gene of the H9N2 avian influenza virus, as detailed in Table 2. A standard curve assay was conducted to confirm the high linearity of the reaction (R² ≥ 0.90). The standard curve for the H9 gene-specific reactions was generated using RNA extracted from a virus stock of A/chicken/Egypt/v1896/2022 (H9N2) at a concentration of 10⁶ EID₅₀/ml, which was serially diluted tenfold from 10⁻¹ to 10⁻⁶. Each dilution was tested in triplicate to evaluate sensitivity and accuracy. The standard curves were created by plotting the quantification cycle values against the log₁₀ of the EID₅₀.

**Table 2:** Oligonucleotide primers used for the amplification of the avian influenza (AIV), (H9N2) HA- protein

Virus - gene	Primer/probe sequence 5’−3’
**Avian Influenza (H9N2)** **HA gene**	**Forward primer**: AGA CGA ATC/T TGT ACA CAA **Reverse primer**: CATGGAGCAATTAGATTCC **Probe -**FAM- AACCGACACAACAACAAGCATAACA-TAMRA

### AIV (H9N2) serology

The hemagglutination inhibition (HI) test was conducted utilizing 4 hemagglutinin (HA) units from avian influenza virus (AIV) with two specific antigens: A/chicken/Egypt//FAO-S33/2021 (H9N2) and A/chicken/Egypt/v1896/2022 (H9N2). In this procedure, two-fold serial dilutions of 25 µl of inactivated chicken serum were prepared in phosphate-buffered saline, followed by the addition of a constant quantity of antigen containing 4 HA units. After a 15-minute incubation period, 25 µl of 1% chicken red blood cells was introduced, and the HI titer was determined based on the last well exhibiting hemagglutination inhibition. The titers were reported as log_2_ geometric mean titres (GMT).

### Histopathology 

Lung and tracheal samples were collected for histopathological study. Specimens from each group were fixed at 10% neutral buffered formalin for 48 h. Following routine processing of the fixed tissues, they were sectioned at 5 microns thick and stained with hematoxylin and eosin (Suvarna et al. 2018). Histological lesions were assessed, assigned scores, and captured through photomicrography using a light microscope equipped with an Amscope digital camera.

Lesions in trachea and lungs were evaluated using a semi-quantitative ordinal system: (–) absent, (+) mild, (++) moderate, (+++) severe (Table 5). The tracheal and lung lesions grading scales were defined as follows:

- Trachea: Inflammation: (+) minimal and focal inflammatory cells infiltration; scattered heterophil and/or mononuclear infiltration often perivascular, (++) moderate and multifocal to coalescing inflammatory cell infiltration, (+++) Severe and diffuse inflammatory cell infiltration; dense aggregates of heterophils and/or mononuclear cells infiltration. Oedema: (+) slight submucosal separation by clear fluid; (++) moderate separation with mild distortion; (+++) marked swelling and expansion. Loss/desquamation of mucosal epithelium and necrosis: (+) focal epithelial loss; (++) widespread disruption; (+++) full-thickness mucosal necrosis. Fibrino-leucocytic exudate: (+) sparse fibrin strands with occasional leukocytes; (++) moderate fibrin layering with abundant cells; (+++) dense fibrino-leukocytic layers. Goblet cell hyperplasia: (+) slight increase over baseline; (++) moderate increase with some clusters; (+++) extensive proliferation with goblet cell crowding. Dilated blood vessels: (+) occasional dilation; (++) multiple dilated vessels with congestion; (+++) prominent dilation with haemorrhage.
- Lungs: Necrosis: (+) focal necrosis in limited parabronchial regions; (++) multifocal necrosis involving multiple parabronchia; (+++) extensive necrosis affecting large regions of the parabronchial network. Cellular infiltrate: (+) localized infiltration; (++) multifocal and moderate infiltration across multiple parabronchial walls and interparabronchial septae; (+++) dense and diffuse infiltration. Edema: (+) slight interstitial fluid; (++) moderate expansion and separation of the interparabronchial connective tissue; (+++) severe interstitial oedema with marked diffuse separation of interparabronchial connective tissue. Fibrino-leucocytic exudate: (+) small aggregates of fibrin and few leukocytes; (++) moderate fibrin deposits and mixed inflammatory cells; (+++) extensive fibrin deposition densely packed with leukocytes.

### Assessment of the inactivated AIV vaccine based on strain A/chicken/Egypt//FAO-S33/2021 (H9N2) serological responses following commercial layers prime-boost immunization protocol

A field trial was carried out in commercial facilities housing 20,000 DEKLB layer chickens. The study encompassed three separate houses located on the same premises, with each house containing bird populations between 6,000 and 6,600. All chickens received a subcutaneous injection of the inactivated vaccine PF/2021 in the neck, administered at a dosage of 0.5 ml when the birds were 23 days old, followed by a booster at 58 days. Thirty birds were randomly selected from different areas in each poultry house then labelled. Blood samples were subsequently collected at various intervals: 7, 23, 44, 58, 79, and 110 days of age. The aforementioned was performed to assess the serological response to the homologous antigen A/chicken/Egypt//FAO-S33/2021 (H9N2). The sample size of 30 birds was selected from a population of 6000–6600 for antibody evaluation based on considerations of statistical power, precision, and logistical feasibility with a specified confidence level (95%).

### Statistical analysis

For the serological analyses, results are presented as mean ± SEM (Standard Error of Mean) and the data were analysed using one-way analysis of variance (ANOVA). Tukey’s Honestly Significant Difference (HSD) test was used as a post hoc analysis with statistical significance established at *p* < 0.05. The clinical indices were calculated according to (Grund et al. 2014). The respiraoty viral shedding were assessed in comparison to the control sham vaccinated and infected group (Group E) using a two-way ANOVA, with Dunnett’s multiple comparison test as a post hoc analysis at *p* < 0.05. GraphPad Prism version 8 for Windows, developed by GraphPad Software in La Jolla, California, USA, was utilized for data visualization.

### Phylogenetic analysis

The amino acid alignment was conduct for 35 AIV (H9N2) strains using CLUSTALW multiple sequence alignment algorithm. The evolutionary history was inferred by using the Maximum Likelihood method and JTT matrix-based model (Jones et al. 1992). Evolutionary analyses were conducted in MEGA11 and 500 bootstrap replicates were calculated (Tamura et al. 2021).

## Results

### Phylogenetic analysis of the hemagglutinin protein from the AIV vaccine strain A/chicken/Egypt//FAO-S33/2021 (H9N2)

**Fig. 1:**
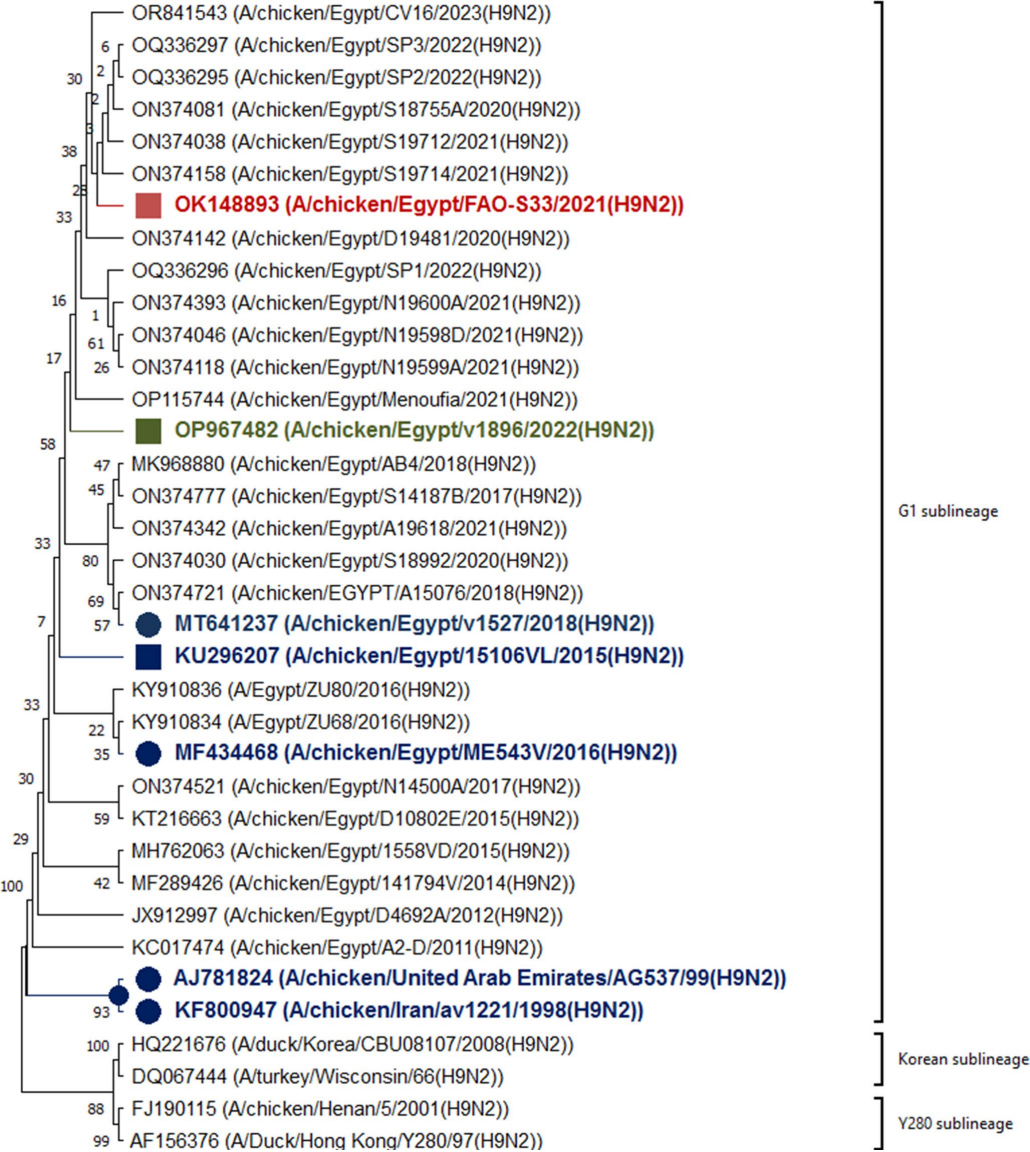
Evolutionary analysis of the hemmaggluting protein of the AIV (H9N2) selected reference strains. The evolutionary history was inferred by using the Maximum Likelihood method and JTT matrix-based model (Jones et al. 1992). The tree with the highest log likelihood (−1813.83) is shown. The percentage of trees in which the associated taxa clustered together is shown next to the branches. Initial tree(s) for the heuristic search were obtained automatically by applying Neighbor-Join and BioNJ algorithms to a matrix of pairwise distances estimated using the JTT model, and then selecting the topology with superior log likelihood value. A discrete Gamma distribution was used to model evolutionary rate differences among sites (3 categories (+ G, parameter = 0.3825). This analysis involved 36 amino acid sequences. All positions containing gaps and missing data were eliminated (complete deletion option). There were a total of 265 positions in the final dataset. Evolutionary analyses were conducted in MEGA11 (Tamura et al. 2021). Red-labelled taxa refer to the seed strain of the used vaccine, whereas green-labelled taxa refer to the challenge virus used in the experimental studies and as antigen in the hemagglutination-inhibition test. Blue-labelled taxa refer to seed strains of other inactivated AIV H9N2 commercial vaccines

In this research, a diverse selection of H9 vaccine seed strains and circulating H9 field strains was utilized to assess the phylogenetic relationships between the administered vaccine and other commercially available vaccines. The comparison were set in relation to the circulating field viruses. The phylogenetic analysis of the hemagglutinin (HA) protein from the H9 vaccine seed strains revealed that the A/chicken/Egypt//FAO-S33/2021 (H9N2) exhibited a 99.44% amino acid similarity to the H9N2 strain isolated in 2023 (A/chicken/Egypt/CV16/2023(H9N2)). Notably, the vaccine seed strain exhibits over 99% genetic identity with the AIV (H9N2) strains from 2021 to 2023 and is phylogenetically grouped within the same subclade (Fig. 1). In contrast, other vaccine strains demonstrated a similarity relatedness ranging from 97.48% to 98.14% with the strains from 2021 to 2023.

### Study 1: PF vaccine generate AI-H9N2 HI specific antibodies predictive for protection against challenges with the recent AIV (H9N2) isolated strains

**Fig. 2:**
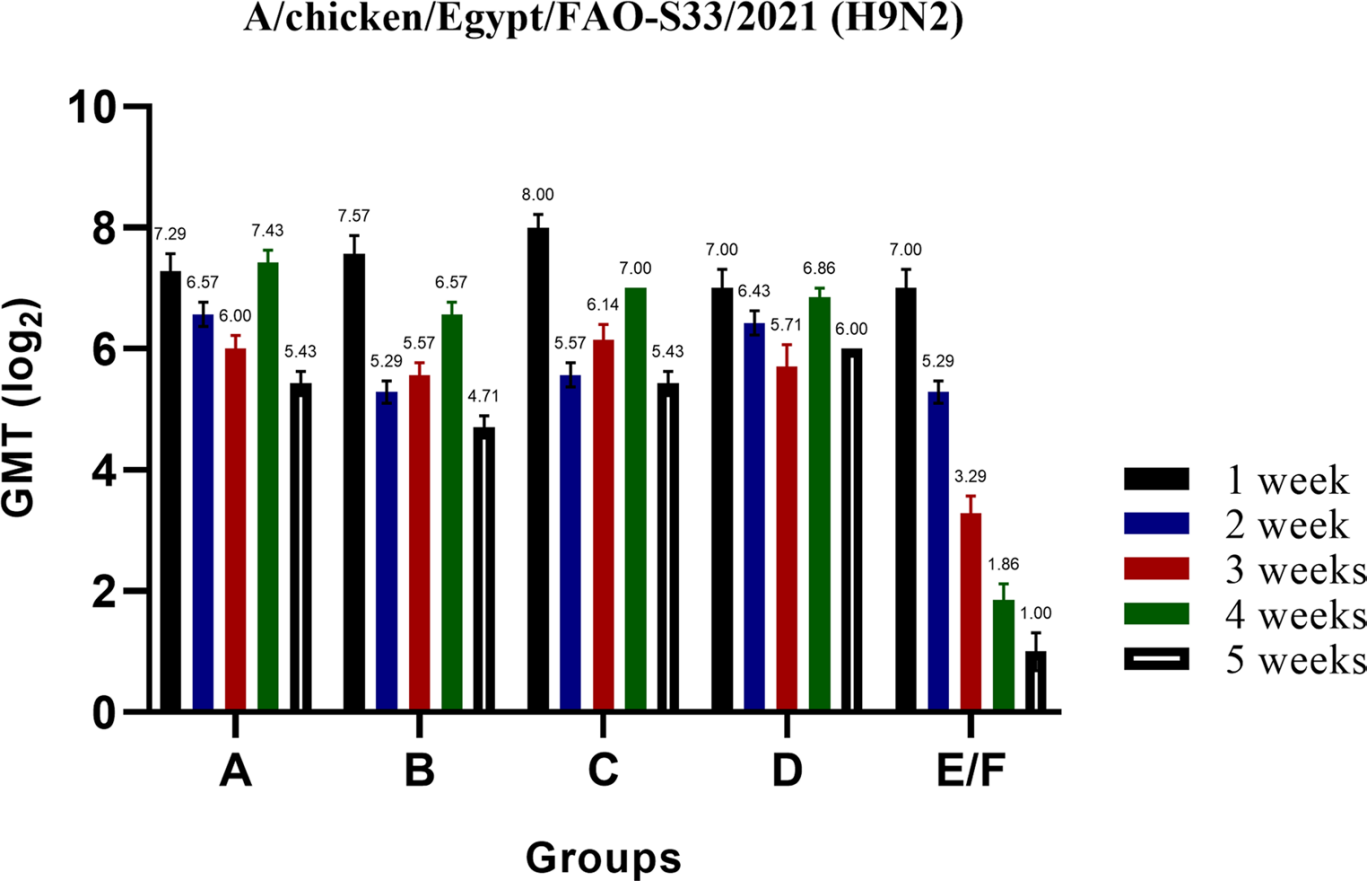
Serology of the experimental birds as determined by the HI test. Serological responses of birds vaccinated with different doses of inactivated Avian Influenza strain (AIV), strain A/chicken/Egypt//FAO-S33/2021 (H9N2) at different ages, measured using HI test to determine the level of specific antibodies against the applied vaccine in the different experimental groups. The titres are expressed as HI mean titers (log2 ± SE) using 4 HA units of the AI H9N2 antigen (A/chicken/Egypt//FAO-S33/2021 (H9N2)). The four groups of vaccinated birds, designated as A, B, C, and D, received vaccinations of 0.3 cc/bird at zero day of age, 0.3 cc/bird at three days of age, 0.5 cc/bird at three days of age, and 0.5 cc/bird at seven days of age, respectively. A fifth group served as the control and was sham-vaccinated and designated as E/F

**Fig. 3:**
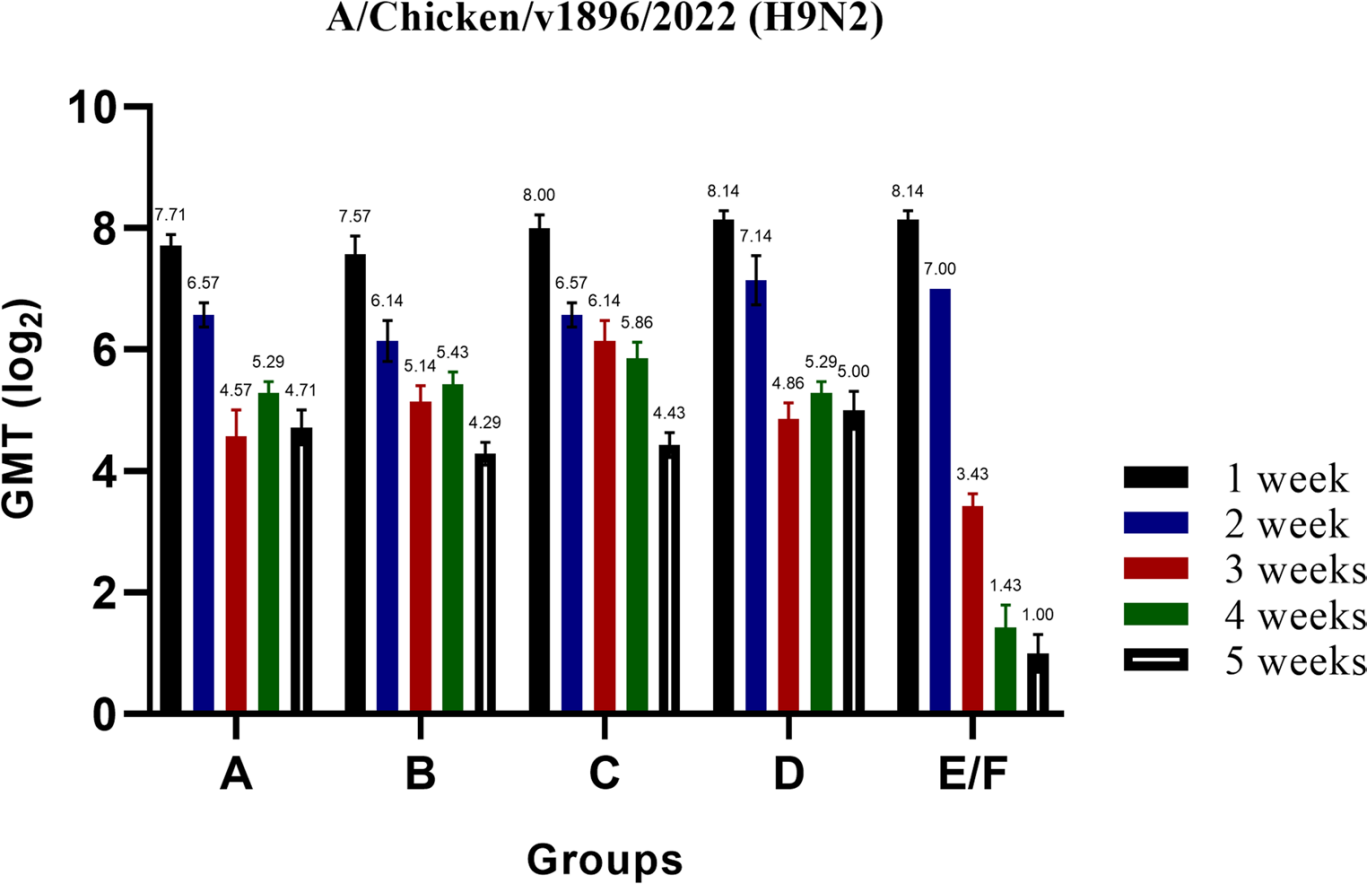
Serology of the experimental birds as determined by the HI test. Serological responses of birds vaccinated with different doses of inactivated Avian Influenza strain (AIV), strain A/chicken/Egypt//FAO-S33/2021 (H9N2) at different ages, measured using HI test to determine the level of specific antibodies against the applied vaccine in the different experimental groups. The titres are expressed as HI mean titres (log2 ± SE) using 4 HA units of the AI H9N2 antigen (A/chicken/Egypt/v1896/2022 (H9N2)). The four groups of vaccinated birds, designated as A, B, C, and D, received vaccinations of 0.3 cc/bird at zero day of age, 0.3 cc/bird at three days of age, 0.5 cc/bird at three days of age, and 0.5 cc/bird at seven days of age, respectively. A fifth group served as the control and was sham-vaccinated and designated as E/F

To assess the immunogenicity of the inactivated avian influenza virus (AIV) vaccine, designated as PF vaccine (A/chicken/Egypt//FAO-S33/2021 (H9N2)), various vaccination strategies were implemented. Serum samples were obtained on a weekly basis, and the HI-Ab responses to the AI (H9N2) were evaluated using two distinct antigens: A/chicken/Egypt//FAO-S33/2021 (H9N2) as the autologous antigen (Fig. 2; Table 3) and A/chicken/Egypt/v1896/2022 as a predictive measure for the virus challenge (Fig. 3; Table 4). The hemagglutination Inhibition titres of one-day-old commercial broilers, reflecting maternal derived antibodies (MDA), were measured at 8.9 ± 0.1 log_2_ for A/chicken/Egypt/FAO-S33/2021 and 9.3 ± 0.5 log_2_ for A/chicken/Egypt/v1896/2022. As depicted in Figs. 2 and 3; Tables 3 and 4, administering a single dose of the inactivated PF (H9N2) vaccine at zero day of age (Group A) led to a decline in MDA, which became evident by the time the birds reached two weeks of age. The geometric mean antibody titres showed an increase from 2 to 4 weeks of age, ranging from 6.57 ± 0.20 log_2_ to 7.43 ± 0.2 log_2_ for A/chicken/Egypt/FAO-S33/2021, and from 6.57 ± 0.20 log_2_ to 5.29 ± 0.18 log_2_ for A/chicken/Egypt/v1896/2022 Thereafter, the titres declined at the fifth week of age to 5.43 ± 0.20 log_2_ or 4.71 ± 0.29 log_2_, when used either A/chicken/Egypt/FAO-S33/2021 or A/chicken/Egypt/v1896/2022, respectively (Figs. 2 and 3; Tables 3 and 4).

**Table 3:** *Serological monitoring of avian influenza HI-Ab responses in chickens immunized with different vaccination strategies using an inactivated AIV vaccine, strain A/chicken/Egypt//FAO-S33/2021 (H9N2) at various ages

Age	Group A	Group B	Group C	Group D	Group E/F
First Week	7.29 ± 0.29^ab^	7.57 ± 0.30^ab^	8.00 ± 0.22^a^	7.00 ± 0.31^b^	7.00 ± 0.31^b^
Second Week	6.57 ± 0.20^a^	5.29 ± 0.18^c^	5.57 ± 0.20^bc^	6.43 ± 0.20^ab^	5.29 ± 0.18^c^
Third Week	6.00 ± 0.22^a^	5.57 ± 0.20^a^	6.14 ± 0.26^a^	5.71 ± 0.36^a^	3.29 ± 0.29^b^
Fourth Week	7.43 ± 0.20^a^	6.57 ± 0.20^a^	7.00 ± 0.00^a^	6.86 ± 0.14^a^	1.86 ± 0.26^b^
Fifth Week	5.43 ± 0.20^ab^	4.71 ± 0.18^b^	5.43 ± 0.20^ab^	6.00 ± 0.30^a^	1.00 ± 0.31^c^

*HI mean titers (log_2_ ± SE) using 4 HA units of the the AI H9N2 antigen (A/chicken/Egypt//FAO-S33/2021 (H9N2))

- Different superscript letters (a, b & c) in the same raw indicate a significant difference between the value of the geometric mean titers at the same time point with a (*P* ≤ 0.05)

Single injection with half dose of the inactivated PF (H9N2) vaccine at 3 days old (Gp B) led to increasing in the HI-titres from 2 to 4 weeks of age ranging from 5.29 ± 0.18 log_2_ to 6.57 ± 0.20 log_2_ against the A/chicken/Egypt/FAO-S33/2021 antigen or 6.14 ± 0.34 to 5.43 ± 0.20 log_2_ against the A/chicken/Egypt/v1896/2022. Thereafter, the titres declined to 4.7 ± 0.18 log_2_ or 4.29 ± 0.18 log_2_ when used either A/chicken/Egypt/FAO-S33/2021 or A/chicken/Egypt/v1896/2022, respectively. When used the full dose of the inactivated PF (H9N2) vaccine at 3 days old, increases in the GMTs from 2 to 4 weeks of age ranging from 5.57 ± 0.20 log_2_ to 7.00 ± 0.00 log_2_ against (A/chicken/Egypt/FAO-S33/2021) and 6.57 ± 0.20 log_2_ to 5.86 ± 0.26 log_2_ against the more recent antigen (A/chicken/Egypt/v1896/2022). The titres decreased slightly at 5 week of age to 5.428 ± 0.202 log_2_ or 4.428 ± 0.202 log_2_ against either A/chicken/Egypt/FAO-S33/2021 or A/chicken/Egypt/v1896/2022, respectively (Figs. 2 and 3; Tables 3 and 4).

**Table 4:** *Serological monitoring of avian influenza HI-Ab responses in chickens immunized with different vaccination strategies using an inactivated AIV vaccine, strain A/chicken/Egypt//FAO-S33/2021 (H9N2) at various ages

Age	Group A	Group B	Group C	Group D	Group E/F
Zero Day					
First Week	7.71 ± 0.18	7.57 ± 0.30	8.00 ± 0.22	8.14 ± 0.31	8.14 ± 0.31
Second Week	6.57 ± 0.20	6.14 ± 0.34	6.57 ± 0.20	7.14 ± 0.40	7.00 ± 0.00
Third Week	4.57 ± 0.43^b^	5.14 ± 0.26^ab^	6.14 ± 0.34^a^	4.86 ± 0.26^b^	3.43 ± 0.20^c^
Fourth Week	5.29 ± 0.18^a^	5.43 ± 0.20^a^	5.86 ± 0.26^a^	5.29 ± 0.18^a^	1.43 ± 0.37^b^
Fifth Week	4.71 ± 0.29^a^	4.29 ± 0.18^a^	4.43 ± 0.20^a^	5.00 ± 0.31^a^	1.00 ± 0.31^b^

*HI mean titers (log_2_ ± SE) using 4 HA units of the the AI H9N2 antigen (A/chicken/Egypt/v1896/2022 (H9N2))

- Different superscript letters (a, b & c) in the same raw indicate a significant difference between the value of the geometric mean titers at the same time point with a (*P* ≤ 0.05)

In the case of a one-week-old vaccination, the inactivated PF (H9N2) vaccine resulted in interference with maternal-derived antibodies (MDA) at 21 days of age. This interference followed by increasing in the GMTs to approximately 6 log_2_ against A/chicken/Egypt/FAO-S33/2021 and 5 log_2_ against A/chicken/Egypt/v1896/2022 by 4 to 5 weeks of age. However, all applied vaccination strategies against AIV (H9N2) led to statistically significant increases in the HI-Ab GMTs (*P* ≤ 0.05) at the third, fourth and fifth weeks of age compared to the non-vaccinated group (Tables 2 and 3).

### Study 2: Clinical protection of the different dosages of the PF (H9N2) vaccine derived from AIV (A/chicken/Egypt//FAO-S33/2021 (H9N2)) strain against challenges at either 14 or 21 days old with (A/chicken/Egypt/v1896/2022 (H9N2))

- **14 days old challenge**.

**Fig. 4:**
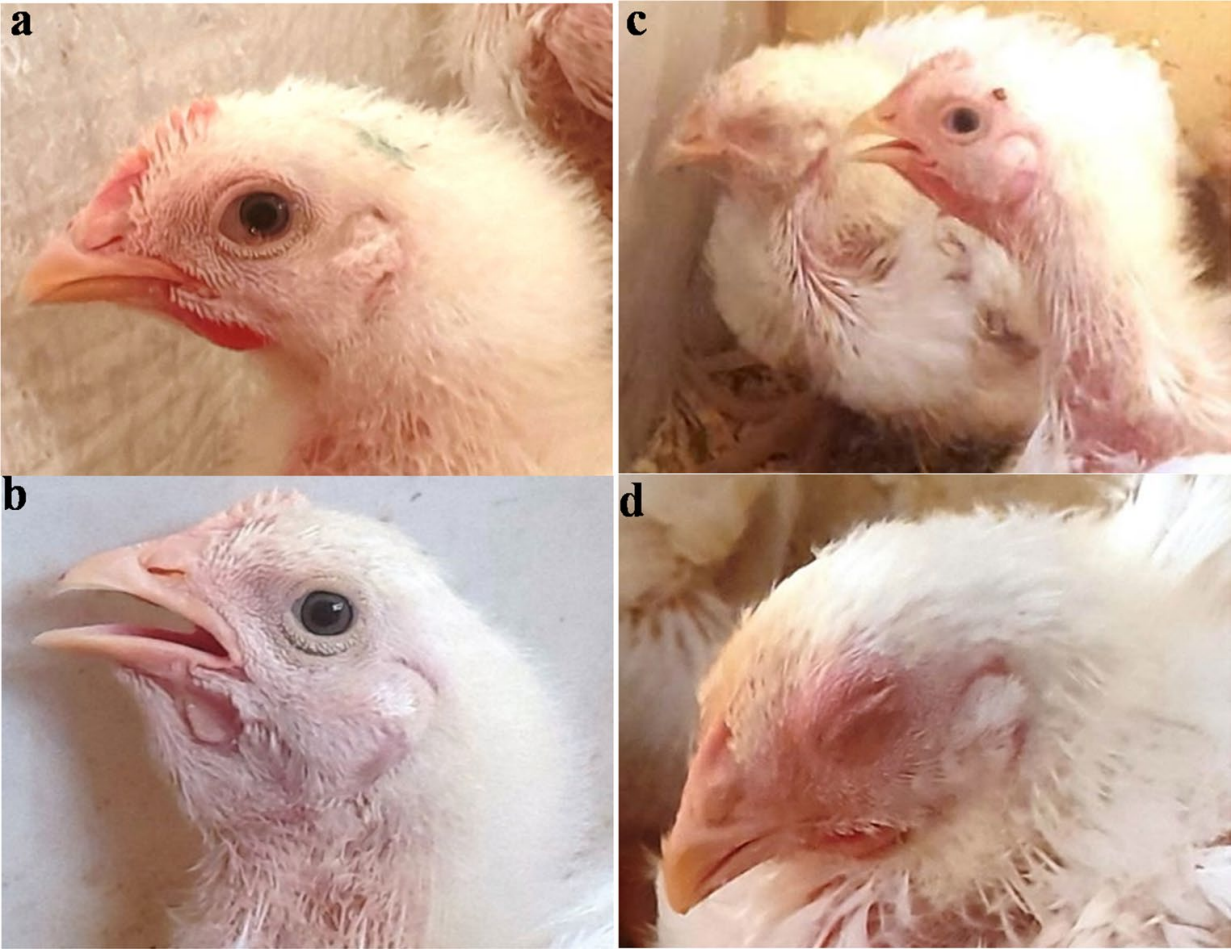
Clinical observation of the experimentally infected birds post challenge with avian influenza virus (A/chicken/Egypt/v1896/2022 (H9N2)). Challenged non-vaccinated broiler chicken showed: **a**- Conjunctivitis with ocular discharge at 5 dpi (14 days challenge). **B**- Dyspnoea with respiratory distress at 6 dpi (14 days challenge). **c**- Dyspnoea with sinusitis at 5 dpi (21 days challenge).** d** - Depressed, inactive with conjunctivitis at 7 dpi (21 days challenge)

**Fig. 5:**
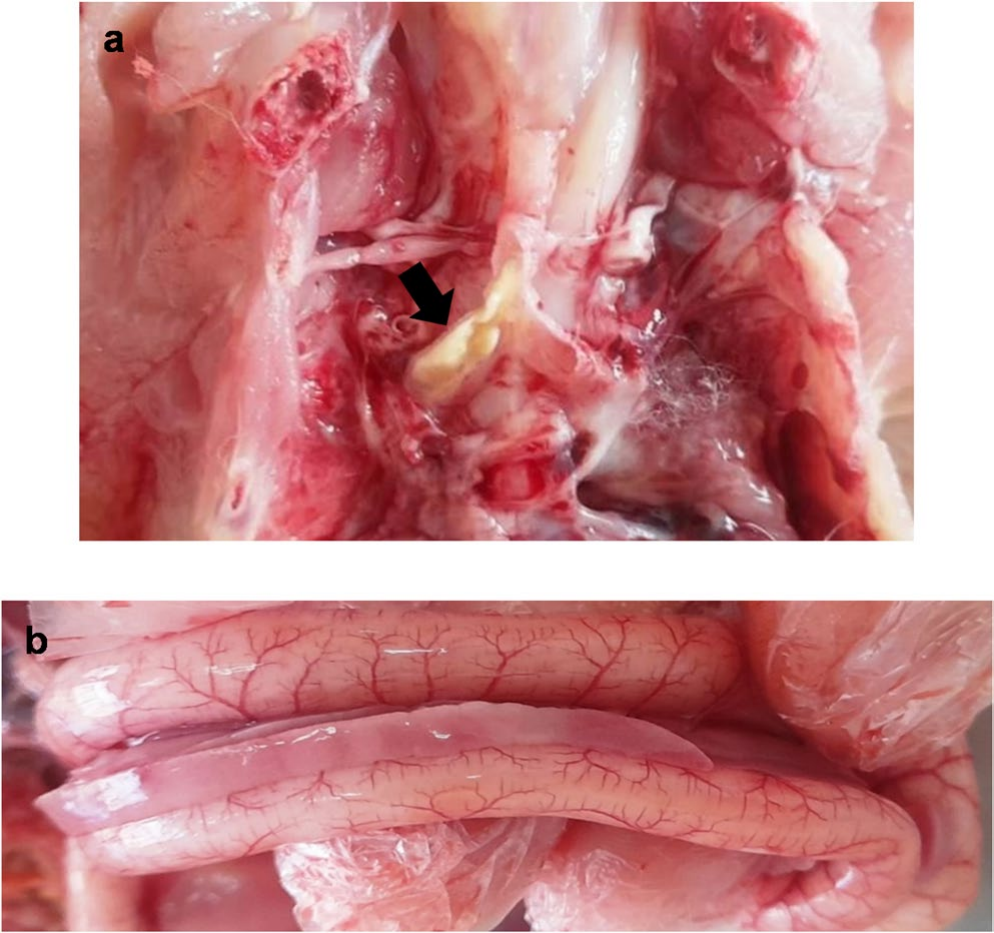
Pathological lesions in dead birds following challenge with avian influenza virus (A/chicken/Egypt/v1896/2022 (H9N2)). **a**- Fibrino-necrotic cast formation at the tracheal bifurcation (arrow) in challenged non-vaccinated broiler chicken at 7 dpi (21 day challenge). **b**- Haemorrhagic pancreatitis (arrow) in challenged non-vaccinated broiler chicken at 7 dpi (21 day challenge)

**Fig. 6:**
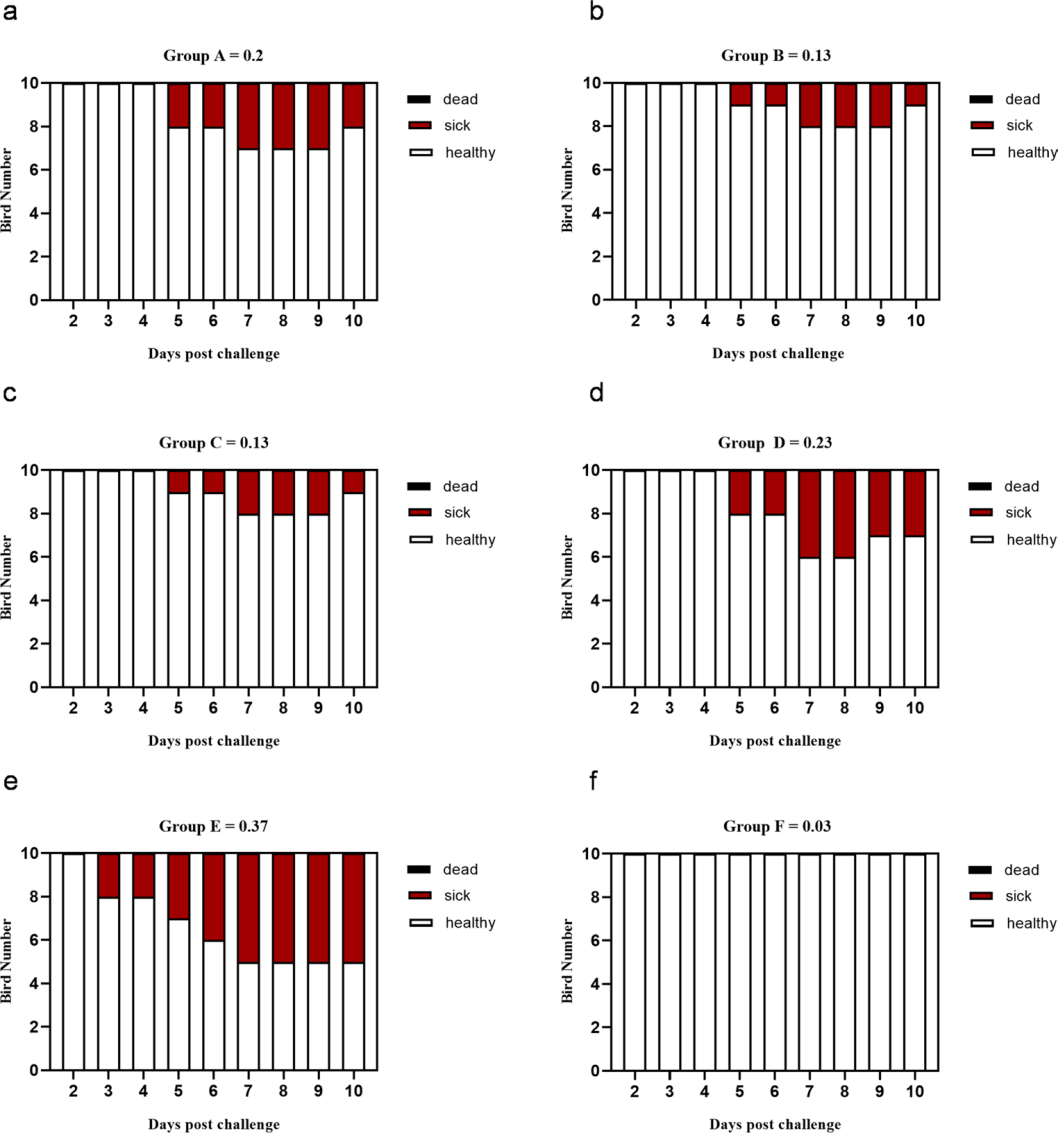
**(a-f)** Avian influenza virus (A/chicken/Egypt/v1896/2022 (H9N2) clinical course and score in the experimental groups in the case of 14 days old challenge. Broiler chickens with MDAs were vaccinated with different doses of inactivated Avian Influenza strain (AIV), strain A/chicken/Egypt//FAO-S33/2021 (H9N2) at different ages and were challenged with 10^8^/EID_50_/0.1 ml per bird of avian influenza virus (A/chicken/Egypt/v1896/2022 (H9N2) at 14 days old. The birds were observed for clinical signs with calculation of clinical indices according to (Grund et al. 2014) and graphed using GraphPad Prism version 8. Groups A, B, C, and D, received vaccinations of 0.3 cc/bird at zero day of age, 0.3 cc/bird at three days of age, 0.5 cc/bird at three days of age, and 0.5 cc/bird at seven days of age, respectively followed by 14 days old challenge. Group E and F were sham vaccinated either infected with (A/chicken/Egypt/v1896/2022 (H9N2) at 14 days of age or not, respectively

As depicted in Figs. 4, 5 and 6(a-f), the effects of an intraocular challenge with 10^6^ EID_50_/0.1 ml of the A/chicken/Egypt/v1896/2022 (H9N2) virus, belonging to G1 sublineage, were evaluated in 14-day-old broiler chickens. Notably, non-vaccinated birds began exhibiting clinical symptoms including conjunctivitis, depression, ocular discharge, sneezing, and frequent gasping as early as three dpc (Fig. 4). These clinical signs persisted for up to ten dpc, resulting in a clinical index of 0.37 (Fig. 6e). The pathological lesions corresponded with the clinical signs in the non-vaccinated birds such as fibrino-necrotic cast formation at the tracheal bifurcation and haemorrhagic pancreatitis at 7 dpc.

In contrast, when either vaccination was administered with the PF (H9N2) at three days of age, at half or full dosage per bird, clinical signs emerged on the fifth day post-challenge, with the vaccinated groups demonstrating a lower clinical index of approximately 0.13 for both dosage levels (Fig. 6b-c). Conversely, groups vaccinated at zero day and seven days of age exhibited clinical signs by the fifth day post-challenge, with clinical index of 0.2 and 0.23, respectively (Fig. 6d-e).

**Fig. 7:**
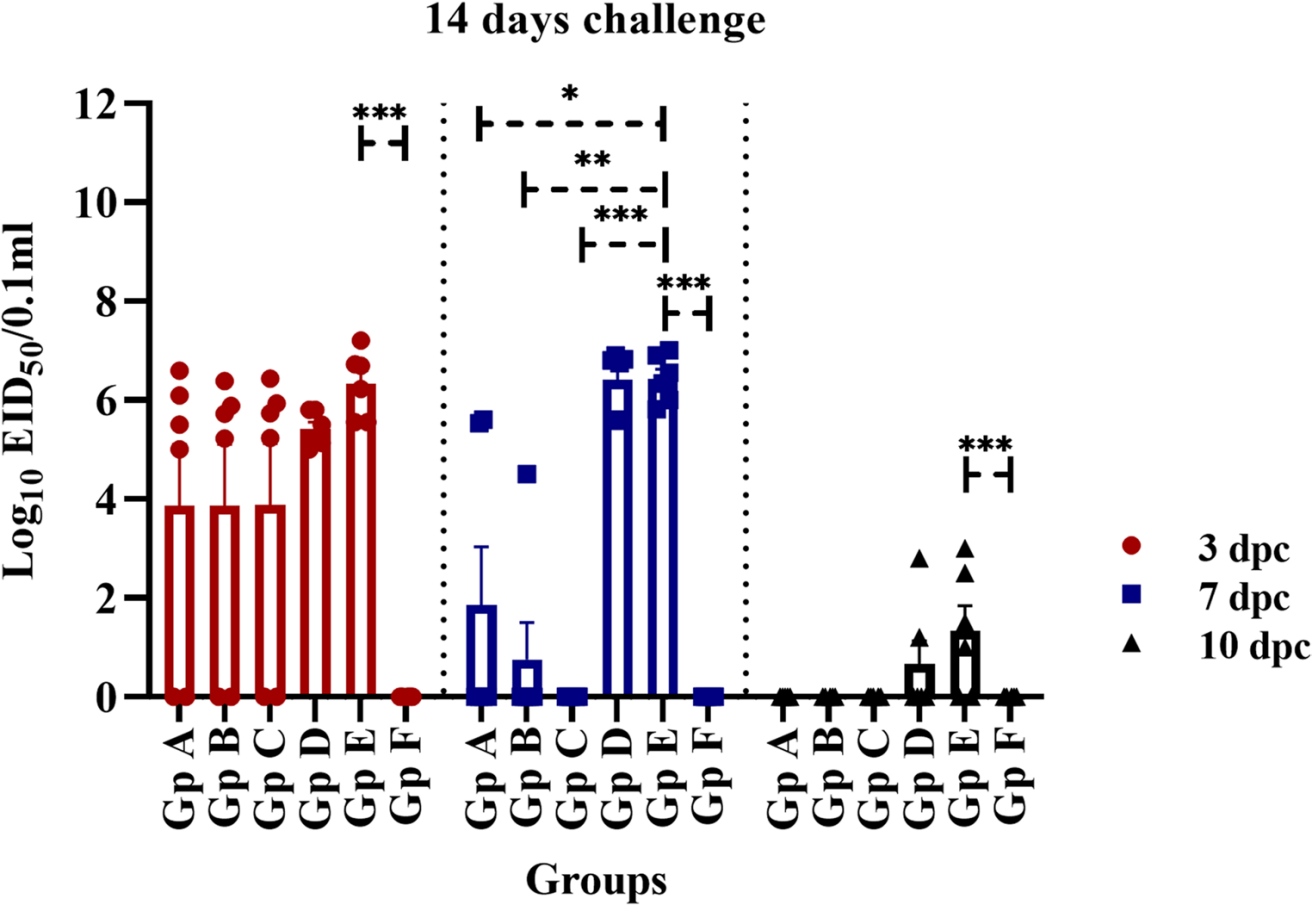
Avian influenza virus (H9N2) shedding in birds vaccinated with different doses of inactivated Avian Influenza strain (AIV), strain A/chicken/Egypt//FAO-S33/2021 (H9N2) at different ages and were challenged with 10^8^/EID_50_/0.1 ml per bird of avian influenza virus (A/chicken/Egypt/v1896/2022 (H9N2) at 14 days old. Scatter plots of tracheal shedding from vaccinated and sham control non-vaccinated birds at 3, 7 and 10 dpc with avian influenza virus (A/chicken/Egypt/v1896/2022 (H9N2). Shedding titres are expressed as log_10_ with error bars included. The limit of detection of the qRRT-PCR was 2log_10_ EID_50_/0.1 ml. The data were graphed using GraphPad Prism version 8. Groups A, B, C, and D, received vaccinations of 0.3 cc/bird at zero day of age, 0.3 cc/bird at three days of age, 0.5 cc/bird at three days of age, and 0.5 cc/bird at seven days of age, respectively followed by 14 days old challenge. Group E and F were sham vaccinated either infected with (A/chicken/Egypt/v1896/2022 (H9N2)) at 14 days of age or not, respectively

The viral genome copies during AIV (H9N2) infection were evaluated through quantitative real-time RT-PCR analysis of tracheal swabs collected from both vaccinated and non-vaccinated birds at various post-infection time points. As illustrated in Fig. 7, control birds (Gp E) that received a sham vaccination exhibited high levels of viral shedding in the trachea, with all six birds showing a mean viral titre of 10^6.3^ EID_50_/0.1 ml at three dpc with statistically significant difference (*P* ≤ 0.001) from non-infected non vaccinated group (Gp F). In contrast, birds that received a single immunization with the inactivated PF (H9N2) vaccine at either zero or three days of age demonstrated reduced viral shedding, with 4/6 birds shedding a mean of 10^3.9^ EID_50_/0.1 ml at the same time point. Notably, birds immunized with the inactivated PF (H9N2) vaccine at seven days of age exhibited the highest levels of viral shedding among the vaccinated group, with all six birds shedding a mean of 10^5.4^ EID_50_/0.1 ml at three dpc. By seven days post-challenge, both the sham vaccinated control group and the birds vaccinated at seven days old continued to shed elevated viral titres from the trachea, with a mean of 10^6.4^ EID_50_/0.1 ml observed in all six birds. Conversely, those immunized at zero day of age showed a significant reduction in viral shedding, with only 2/6 birds shedding, and these two birds had approximately 10^5.5^ EID_50_/0.1 ml of virus with statistically significant difference (*P* ≤ 0.05) from infected non-vaccinated group (Gp E). Additionally, immunization with half dose at three days of age resulted in minimal shedding (mean of 10^0.7^ EID_50_/0.1 ml), with only 1/6 birds shedding a10^4.5^ EID_50_/0.1 ml at seven dpc with statistically significant difference (*P* ≤ 0.01) from infected non-vaccinated group (Gp E). Negative detection of AIV (H9N2) genome in all analysed birds vaccinated at 3 days old with the full vaccine dose with statistically significant difference (*P* ≤ 0.001) from infected non-vaccinated group (Gp E). By ten days post-challenge, except for 7 days old vaccination, the virus was no longer detectable in the trachea. However, the sham vaccinated control group still exhibited low levels of viral shedding, with 2/6 shedders and a mean of less than 10^2.0^ EID_50_/0.1 ml viral tracheal shedding.

In order to assess the effectiveness of the vaccine under investigation in inhibiting virus transmission among non-immunized birds, three non-H9N2 immunized birds (with MDA of HI GMT ˂ 2.00 log_2_) were introduced into the various vaccinated groups, three days after a 14-days old challenge. Swabs were obtained from the contact birds seven days post-introduction. Within the control group, which did not receive the H9N2 vaccination, 2 out of 3 birds were observed to shed the virus, with titres measuring 10^5.3^ EID_50_/0.1 ml. In contrast, in all vaccinated cohorts, only 1 out of 3 birds exhibited viral shedding, with titers ranging from 10^4.5 to 5^ EID_50_/0.1 ml from the trachea, seven days after contact.

Histopathological examination of the tracheal sections from non-challenged, non-vaccinated chicks showed the normal histologic tracheal architecture (Fig. 10f_1, 2_). While, those non-vaccinated but H9N2 showed severe necrosis and desquamation of the tracheal mucosal, loss of cilia from the non-desquamated cells, with intense inflammatory cell infiltrate and oedema in the lamina propria. In addition fibrinous exudate intermixed with cellular debris were prominent in the tracheal lumen (Fig. 10e_1, 2_). Tracheal sections from chicks vaccinated at day zero and challenged at 14 days of age showed goblet cell hyperplasia, mild desquamation of the lining epithelium with partial loss of cilia and inflammatory infiltrate in the tracheal wall (Fig. 10a_1, 2_). Tracheal section from birds vaccinated at day 3 of age either at half or full dose showed similar histologic lesions consisting of mild loss of mucosal epithelium and cilia, oedema and leucocytic infiltration in the lamina propria (Fig. 10b_1, 2_ and c_1, 2_). Tracheal sections from birds vaccinated at day 7 and challenged at day 14 showed mild leucocytic infiltration, oedema and loss of cilia (Fig. 10d_1, 2_; Table 5).

**Table 5:** Histopathological scoring of lesions in trachea and lungs of experimentally challenged and vaccinated chicks

	Trachea						Lungs				
Groups	Inflammation	Edema	Loss of cilia	Necrosis and desquamation of mucosal epithelium	Fibrino-leucocytic exudate	Goblet cell hyperplasia	Dilated blood vessels	Necrosis	Cellular infiltrate	Edema	Fibrino-leucocytic exudate
14 days-old challenge											
**Group A**	**++**	**+**	**+**	**+**	**-**	**+**	**++**	**-**	**+**	**++**	**++**
**Group B**	**+**	**++**	**+**	**+**	**-**	**+**	**+**	**-**	**++**	**+**	**+**
**Group C**	**+**	**+**	**+**	**-**	**-**	**-**	**++**	**-**	**+**	**+**	**+**
**Group D**	**+**	**+**	**+**	**+**	**-**	**-**	**+++**	**-**	**+**	**+++**	**++**
**Group E**	**+++**	**++**	**++**	**+++**	**++**	**++**	**++**	**+**	**+++**	**++**	**++**
**Group F**	**-**	**-**	**-**	**-**	**-**	**-**	**-**	**-**	**-**	**-**	**-**
**21 days –old challenge**											
**Group A**	**++**	**+**	**++**	**+++**	**-**	**-**	**+**	**-**	**++**	**+**	**+**
**Group B**	**++**	**+**	**++**	**+**	**+**	**+++**	**+**	**-**	**+**	**+**	**+**
**Group C**	**+**	**+**	**+**	**-**	**-**	**++**	**++**	**-**	**+**	**++**	**+**
**Group D**	**++**	**+**	**++**	**++**	**-**	**+**	**+++**	**-**	**++**	**+++**	**++**
**Group E**	**+++**	**++**	**+++**	**+++**	**+++**	**+++**	**++**	**++**	**+++**	**++**	**++**
**Group F**	**-**	**-**	**-**	**-**	**-**	**-**	**-**	**-**	**-**	**-**	**-**

• Trachea: Inflammation: (+) minimal and focal inflammatory cells infiltration; scattered heterophil and/or mononuclear infiltration often perivascular, (++) moderate and multifocal to coalescing inflammatory cell infiltration, (+++) Severe and diffuse inflammatory cell infiltration; dense aggregates of heterophils and/or mononuclear cells infiltration. Oedema: (+) slight submucosal separation by clear fluid; (++) moderate separation with mild distortion; (+++) marked swelling and expansion. Loss/desquamation of mucosal epithelium and necrosis: (+) focal epithelial loss; (++) widespread disruption; (+++) full-thickness mucosal necrosis. Fibrino-leucocytic exudate: (+) sparse fibrin strands with occasional leukocytes; (++) moderate fibrin layering with abundant cells; (+++) dense fibrino-leukocytic layers. Goblet cell hyperplasia: (+) slight increase over baseline; (++) moderate increase with some clusters; (+++) extensive proliferation with goblet cell crowding. Dilated blood vessels: (+) occasional dilation; (++) multiple dilated vessels with congestion; (+++) prominent dilation with haemorrhage

• Lungs: Necrosis: (+) focal necrosis in limited parabronchial regions; (++) multifocal necrosis involving multiple parabronchia; (+++) extensive necrosis affecting large regions of the parabronchial network. Cellular infiltrate: (+) localized infiltration; (++) multifocal and moderate infiltration across multiple parabronchial walls and interparabronchial septae; (+++) dense and diffuse infiltration. Edema: (+) slight interstitial fluid; (++) moderate expansion and separation of the interparabronchial connective tissue; (+++) severe interstitial oedema with marked diffuse separation of interparabronchial connective tissue. Fibrino-leucocytic exudate: (+) small aggregates of fibrin and few leukocytes; (++) moderate fibrin deposits and mixed inflammatory cells; (+++) extensive fibrin deposition densely packed with leukocytes

The examined lung sections from non-vaccinated, non-challenged birds showed normal histological architecture of avian lungs (Fig. 11f) while sections from other experimental conditions showed varying degrees of interstitial pneumonia sometimes with presence of a fibrino-cellular exudate (Fig. 11a - e). Lung sections from birds vaccinated at day zero and challenged at 14 days of age showed mild hypercellularity of the parabronchial walls with presence of fibrin exudate in the parabronchial lumen. Lung sections from birds vaccinated at day 3 of age (half dose) and challenged at day 14 showed relatively patent parabronchi with presence of variable amounts of fibrinocellular exudate in some parabronchial lumina. Lung sections from birds vaccinated at day 3 (full dose) and challenged at 14 days showing scant amount of eosinophilic fibrinous exudate in the parabronchial lumina, congested air capillaries and dilated interlobular blood vessels with mild perivascular oedema. Lung sections from birds vaccinated at day 7 (0.5 ml) and challenged at day 14 showed moderate amount of fibrinous exudate in the parabronchi and marked extension of the interlobular septa by dilated blood vessels and perivascular eosinophilic oedematous fluid. Lung sections from non-vaccinated but H9N2 challenged birds at age 14 days showed marked extension of the parabronchus and air capillaries with inflammatory cells and congestion together with presence of fibrinous exudate in the parabronchial lumen and diffuse expansion of the perivascular interstitium by oedema and inflammatory cells infiltration.

These results suggest that administering the inactivated H9N2 vaccine at three days of age offers the most substantial clinical protection against challenges occurring at 14 days of age.

b. **21 days old challenge**.

**Fig. 8:**
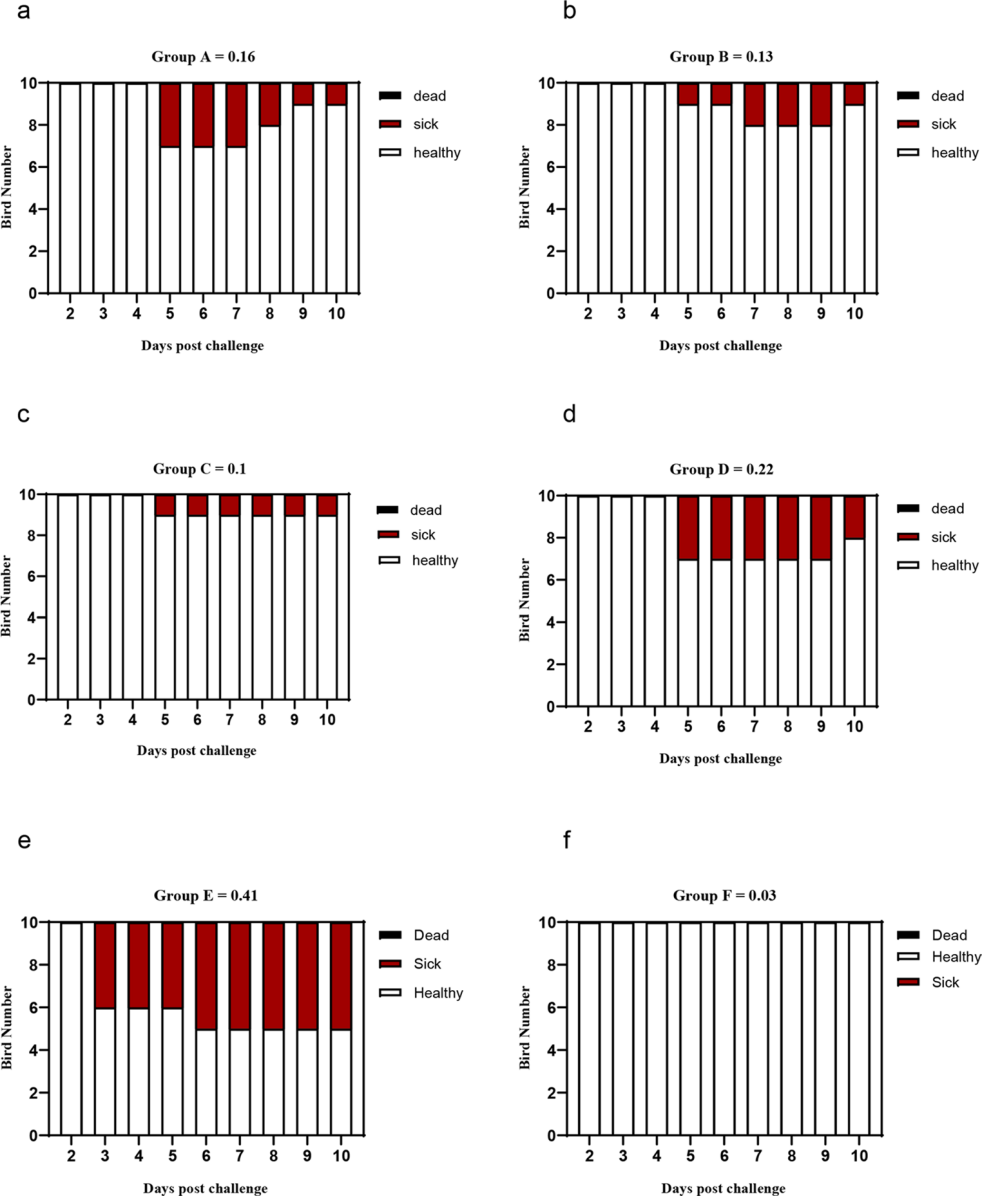
**(a-f)** Avian influenza virus (A/chicken/Egypt/v1896/2022 (H9N2) clinical course and score in the experimental groups in the case of 21 days old challenge. Broiler chickens with MDAs were vaccinated with different doses of inactivated Avian Influenza strain (AIV), strain A/chicken/Egypt//FAO-S33/2021 (H9N2) at different ages and were challenged with 10^8^/EID_50_/0.1 ml per bird of avian influenza virus (A/chicken/Egypt/v1896/2022 (H9N2) at 21 days old. The birds were observed for clinical signs with calculation of clinical indices according to (Grund et al. 2014) and graphed using GraphPad Prism version 8. Groups A, B, C, and D, received vaccinations of 0.3 cc/bird at zero day of age, 0.3 cc/bird at three days of age, 0.5 cc/bird at three days of age, and 0.5 cc/bird at seven days of age, respectively followed by 21 days old challenge. Group E and F were sham vaccinated either infected with (A/chicken/Egypt/v1896/2022 (H9N2) at 21 days of age or not, respectively

The assessment of the recently formulated vaccine based on the moderately recent AIV (A/chicken/Egypt//FAO-S33/2021 (H9N2)) was performed across various dosages and age groups, followed by a challenge with (A/chicken/Egypt/v1896/2022 (H9N2)) at 21 days of age. Observations made after the challenge indicated that all unvaccinated birds displayed clinical signs such as conjunctivitis, lethargy, ocular discharge, sneezing, and, in certain instances, gasping (Fig. 4) accompanied by the development of fibrino-necrotic casts at the tracheal bifurcation and haemorrhagic pancreatitis (Fig. 5). These clinical symptoms persisted in the surviving birds for as long as 10 days post-challenge, leading to a clinical index of 0.41. As depicted in Fig. 8a-f, the lowest clinical index, approximately 0.1, was recorded in birds vaccinated at 3 days of age with a dosage of 0.5 ml per bird. In contrast, the highest clinical index was noted in birds that received a 0.5 ml dosage at 7 days of age.

**Fig. 9:**
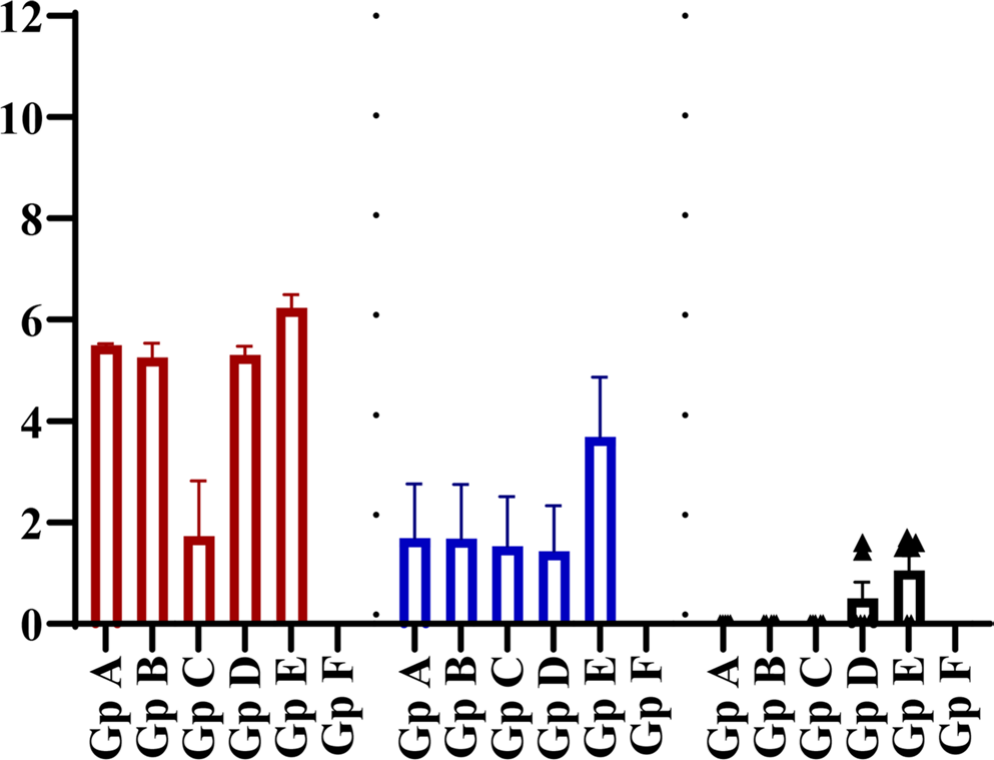
Avian influenza virus (H9N2) shedding in birds vaccinated with different doses of inactivated Avian Influenza strain (AIV), strain A/chicken/Egypt//FAO-S33/2021 (H9N2) at different ages and were challenged with 10^8^/EID_50_/0.1 ml per bird of avian influenza virus (A/chicken/Egypt/v1896/2022 (H9N2) at 21 days old. Scatter plots of tracheal shedding from vaccinated and sham control non-vaccinated birds at 3, 7 and 10 dpc with avian influenza virus (A/chicken/Egypt/v1896/2022 (H9N2). Shedding titres are expressed as log_10_ with error bars included. The limit of detection of the qRRT-PCR was 2log_10_ EID_50_/0.1 ml. The data were graphed using GraphPad Prism version 8. Groups A, B, C, and D, received vaccinations of 0.3 cc/bird at zero day of age, 0.3 cc/bird at three days of age, 0.5 cc/bird at three days of age, and 0.5 cc/bird at seven days of age, respectively followed by 21 days old challenge. Group E and F were sham vaccinated either infected with (A/chicken/Egypt/v1896/2022 (H9N2)) at 21 days of age or not, respectively

As illustrated in Fig. 9, control birds that received sham vaccinations (Gp E) exhibited significant viral shedding in the trachea, with all six birds (6/6) showing high titres of virus (ranging from 10^5.4^ to 10^6.8^ EID_50_/0.1 ml) at 3 dpc with statistically significant difference (*P* ≤ 0.001) from non-infected non vaccinated group (Gp F). Single immunization with the PF (H9N2) vaccine at zero day of age resulted in 100% viral shedding from the trachea (6/6, 10^5.5^ EID_50_/0.1 ml) at the same time point. When the PF (H9N2) vaccine was administered at either three (half dose) or seven days of age, the vaccinated birds demonstrated comparable levels of viral shedding from the trachea at three dpc. Hence, all six birds were tested positive and virus shedding was in the range of 10^4.38^ to 10^5.85^ EID_50_/0.1 ml. Vaccination with full dose at 3 days old led to mean viral tracheal shedding of ˂ 10^5.85^ EID_50_/0.1 ml and 2/6 shedders with statistically significant difference (*P* ≤ 0.001) from infected sham vaccinated group (Gp E). At seven days post-challenge, the sham vaccinated birds (Gp E) continued to shed higher viral titres from the trachea (4/6, mean 10^3.7^ EID_50_/0.1 ml) with statistically significant difference (*P* ≤ 0.001) from non-infected non vaccinated group (Gp F). However, the various vaccinated groups exhibited a reduction of viral shedding from the trachea (2/6, mean 10^˂2^ EID_50_/0.1 ml), with the two shedders exhibiting approximately 10^4.3–5.15^ EID_50_/0.1 ml with statistically significant difference (*P* ≤ 0.05) from infected sham vaccinated group (Gp E). By ten days post-challenge, both vaccinated and sham vaccinated control groups showed negative results for viral detection in the trachea.

The NDV vaccination programs implemented across various experimental groups did not lead to any mortality or tracheal virus shedding in the contact group within the 10 days following contact.

**Fig. 10:**
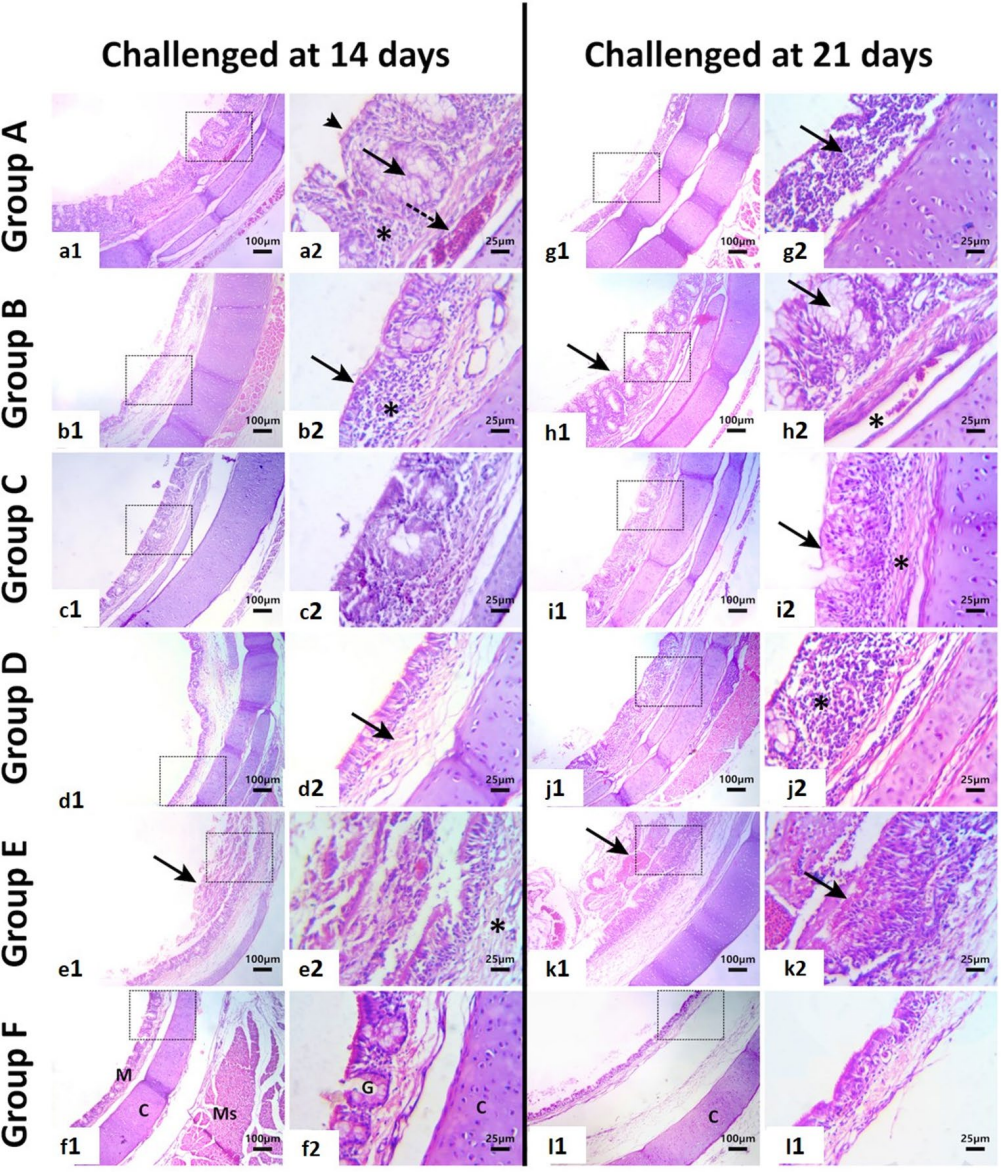
Photomicrograph of H&E-stained tracheal sections from birds of different experimental groups. **a**_**1, 2**_) Tracheal section from birds vaccinated at day zero (0.3 ml) and challenged at 14 days of age showing mild desquamation of the lining epithelium, partial loss of cilia (**arrowhead**), dilated blood vessels **(dashed arrow)**, leucocytic infiltration **(asterisk)** and goblet cell hyperplasia **(arrow)**. **b**_**1, 2**_) Tracheal section from birds vaccinated at day 3 of age and challenged at day 14 showing mild loss of mucosal epithelium and cilia **(arrow)**, edema and leucocytic infiltration in the lamina propria **(asterisk)**. **c**_**1, 2**_) Tracheal section from birds vaccinated at day 3 (0.5 ml) and challenged at 14 days where the mucosa is showing partial loss of cilia from the lining epithelium **(arrow)** moderate leucocytic infiltration and mild edema **(asterisk)**. **d**_**1, 2**_) Tracheal section from birds vaccinated at day 7 (0.5 ml) and challenged at day 14 showing mild leucocytic infiltration **(arrow)**, edema, and loss of cilia. **e**_**1, 2**_) Tracheal section from non-vaccinated but H9N2 challenged birds at age 14 days showing severe necrosis and desquamation of the mucosal epithelium **(arrow)**, partial loss of cilia from the non-desquamated cells, intense inflammatory cell infiltrate and edema **(asterisk)** in the lamina propria. Fibrinous exudate is intermixed with cellular debris in the tracheal lumen. **f**_**1, 2**_ **and l**_**1, 2**_) Tracheal section from non-vaccinated, non-challenged birds showing normal histological architecture of avian trachea with ciliated epithelium in the mucosal layer (M), Mucosal mucus glands (G), cartilaginous (C) and musculature (Ms) layers. **g1**,** 2**) Tracheal section from birds vaccinated at day zero (0.3 ml) and challenged at 21 days of age showing severe necrosis of the mucosal layer **(arrow). h**_**1, 2**_) Tracheal sections from birds vaccinated at day 3 (0.3 ml) and challenged at 21 days showing goblet cell hyperplasia (**arrow)**, leucocytic infiltration, dilated blood vessels **(asterisk)** and edema in the lamina propria. **I**_**1, 2**_**)** Tracheal sections from birds vaccinated at day 3 (0.5 ml) and challenged at 21 days showing goblet cell hyperplasia with a thin layer of mucus covering the mucosa (**arrow)** together with mild leucocytic infiltration and edema in the propria **(asterisk)**. **j**_**1, 2**_) Tracheal sections from birds vaccinated at day 7 (0.5 ml) and challenged at 21 days showing necrosis and loss of the mucosal epithelium with a mononuclear cell infiltrate **(arrow)** and edema. **k**_**1, 2**_) Tracheal section from birds non-vaccinated but challenged at age 21 days showing superficial erosion of the mucosa with adherent layer of fibrin intermixed with cellular debris, inflammatory cells, mucus, and erythrocytes **(arrow)**. The lamina propria shows edema and intense leucocytic infiltration. The scale bar is **100 μm** in all denoted photos **(a-l**_**1**_**)** while **(a-l**_**2**_**)** are higher magnifications with a scale bar **25 μm**. The dashed boxes denote the magnified field

The examined tracheal sections from non-challenged, non-vaccinated chicks showed the normal histologic tracheal architecture (Fig. 10l_1, 2_). While, those non-vaccinated but H9N2 challenged chicks at age 21 days showed severe necrosis and desquamation of the tracheal mucosal, loss of cilia from the non-desquamated cells, with intense inflammatory cell infiltrate and oedema in the lamina propria. In addition, fibrinous exudate intermixed with cellular debris were prominent in the tracheal lumen (Fig. 10k_1, 2_). Tracheal sections from birds vaccinated at day zero and challenged at 21 days of age showed severe necrosis of the mucosal layer (Fig. 10g_1, 2_). However, sections from birds vaccinated at day 3 (half dose) and challenged at 21 days showed goblet cell hyperplasia and inflammation (Fig. 10h_1, 2_). Tracheal sections from birds vaccinated at day 3 (full dose) and challenged at 21 days showed goblet cell hyperplasia with a thin layer of mucus covering the mucosa together with mild leucocytic infiltration and oedema in the propria (Fig. 10i_1, 2_) and (Table 5). Finally, tracheal sections from birds vaccinated at day 7 and challenged at 21 days showed necrosis and loss of the mucosal epithelium with a mononuclear cell infiltrate and oedema (Fig. 10j_1, 2_).

**Fig. 11:**
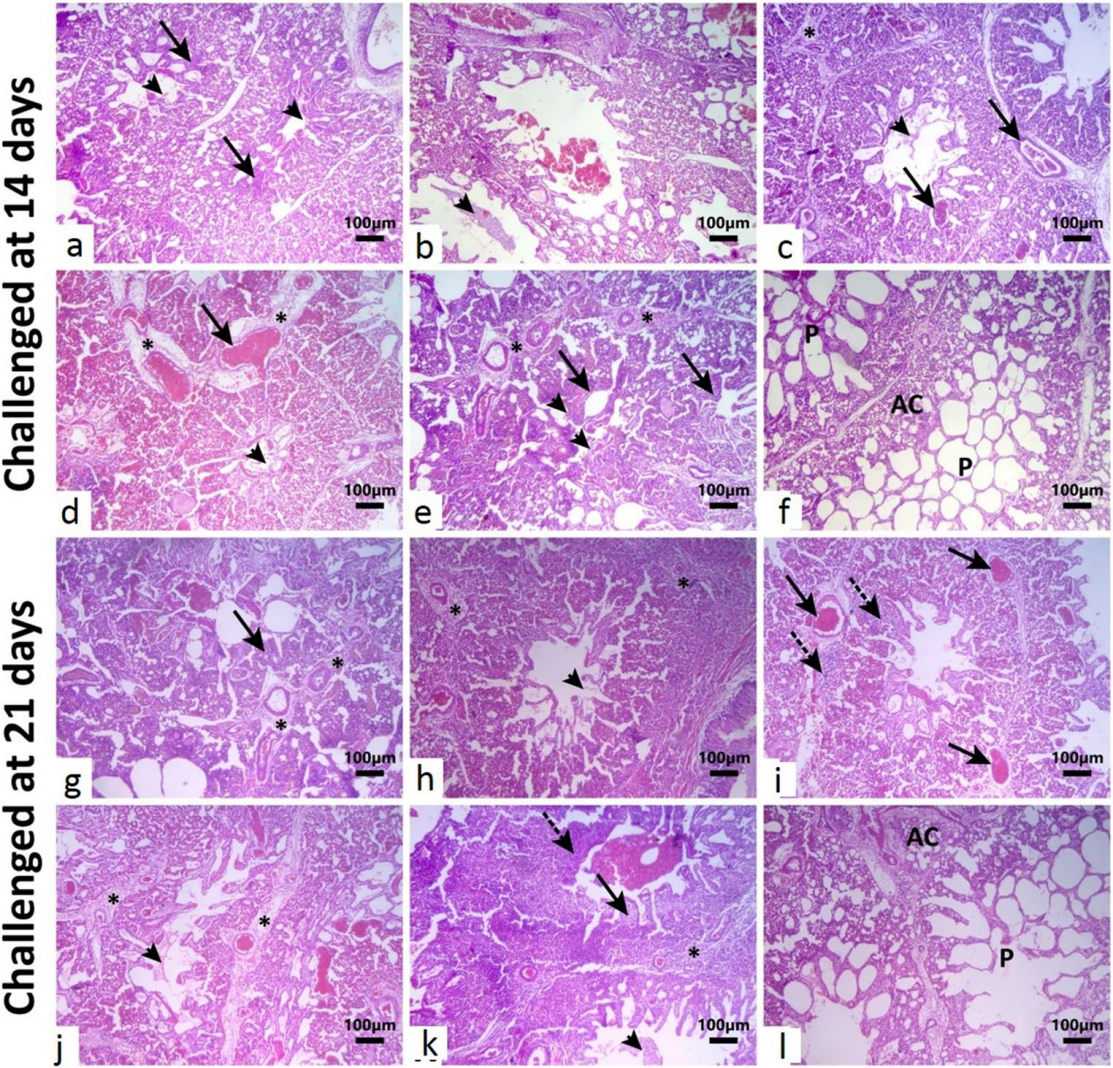
Photomicrograph of H&E-stained lung sections from birds of different experimental groups. **a)** Lung section from birds vaccinated at day zero (0.3 ml) and challenged at 14 days of age showing mild hypercellularity of the parabronchial walls **(arrows)** with presence of scant amount of fibrin exudate in the parabronchial lumen **(arrowheads)**.**b)** Lung section from birds vaccinated at day 3 of age and challenged at day 14 showing relatively patent parabronchi with presence of variable amounts of fibrinocellular exudate in some parabronchial lumina **(arrowhead)**. **c)** Lung section from birds vaccinated at day 3 (0.5 ml) and challenged at 14 days showing scant amount of eosinophilic fibrinous exudate in the parabronchial lumina **(arrowhead)**, congested air capillaries and dilated interlobular blood vessels **(arrows)** with mild perivascular edema **(asterisk)**. **d)** Lung section from birds vaccinated at day 7 (0.5 ml) and challenged at day 14 showing moderate amount of fibrinous exudate in the parabronchi **(arrowhead)** and marked extension of the interlobular septa by dilated blood vessels **(arrow)** and perivascular eosinophilic edematous fluid **(asterisks)**. **e)** Lung section from non-vaccinated but H9N2 challenged birds at age 14 days showing marked extension of the parabronchus and air capillaries with inflammatory cells and congestion **(arrows)** together with presence of moderate amount of fibrin in the parabronchial lumen **(arrowheads)** and diffuse expansion of the perivascular interstitium by edema and moderate inflammatory cells infiltration **(asterisks)**. **f and l**) Lung sections from non-vaccinated, non-challenged birds showing normal histological architecture of avian lungs with patent parabronchi (P) and normal air capillaries (AC). **g)** Lung section from birds vaccinated at day zero and challenged at 21 days of age showing thickening of the parabronchial walls and air capillaries by edema, congestion and cellular infiltrate **(arrow)** with moderate expansion of the interlobular septae by dilated vessels, edema and inflammatory cells **(asterisk)**. **h)** Lung section from birds vaccinated at day 3 (0.3 ml) and challenged at 21 days showing relatively patent parabronchi with presence of scant amount of fibrin in the lumina **(arrowhead)** and moderate interstitial expansion by perivascular edema and cellular infiltrate **(asterisk)**. **i)** Lung section from birds vaccinated at day 3 (0.5 ml) and challenged at 21 days showing dilation of interlobular blood vessels with perivascular and interlobular edema **(arrows)** and mild infiltration of the air capillaries with cellular infiltrate **(dashed arrows). j)** Lung section from birds vaccinated at day 7 (0.5 ml) and challenged at 21 days showing diffuse and marked extension of the interlobular septae with dilated blood vessels, edematous fluid and fibrillar fibrinous material **(asterisk)** and presence of moderate amount of fibrinous exudate in the parabronchi **(arrowhead). k)** Lung section from birds non-vaccinated but challenged at age 21 days showing marked extension of the parabronchial walls and air capillaries by edema and cellular infiltration **(arrow)** with partial necrosis and destruction of the parabronchial wall **(dashed arrow)** and presence of variable amounts of fibrinocellular exudate in the parabronchial lumina. The interlobular septae are edematous and infiltrated with inflammatory cells **(asterisk)**. The Scale bar is **100 μm**

The examined lung sections from non-vaccinated, non-challenged birds showed normal histological architecture of avian lungs (Fig. 11 l). On the other hand, in addition to the presence of fibrinous exudate in the parabronchial lumen and the diffuse expansion of the perivascular interstitium due to edema and inflammatory cell infiltration, lung sections from non-vaccinated but H9N2 challenged birds at age 21 days demonstrated a marked extension of the parabronchus and air capillaries with inflammatory cells and congestion (Fig. 11k). However, sections from other experimental settings had interstitial pneumonia in different degrees, occasionally accompanied by fibrino-cellular exudate (Fig. 11g-j). Lung sections from birds vaccinated at day zero and challenged at 21 days of age showed thickening of the parabronchial walls and air capillaries by oedema, congestion and cellular infiltrate with expansion of the interlobular septae by dilated vessels, oedema and inflammatory cells. Lung sections from birds vaccinated at day 3 (half dose) and challenged at 21 days showed relatively patent parabronchi with presence of scant amount of fibrin in the Lumina and moderate interstitial expansion by perivascular oedema and cellular infiltrate. Lung sections from birds vaccinated at day 3 (full dose) and challenged at 21 days showed dilation of interlobular blood vessels with perivascular and interlobular oedema and mild infiltration of the air capillaries with cellular infiltrate. Lung sections from birds vaccinated at day 7 and challenged at 21 days showed diffuse and marked extension of the interlobular septae with dilated blood vessels, edematous fluid and fibrillar fibrinous material and presence of moderate amount of fibrinous exudate in the parabronchi (Table 5).

Prime and boost vaccination with inactivated PF/2021 vaccine induced protective level of humoral immune response against H9N2 AIV in commercial layer houses.

**Fig. 12:**
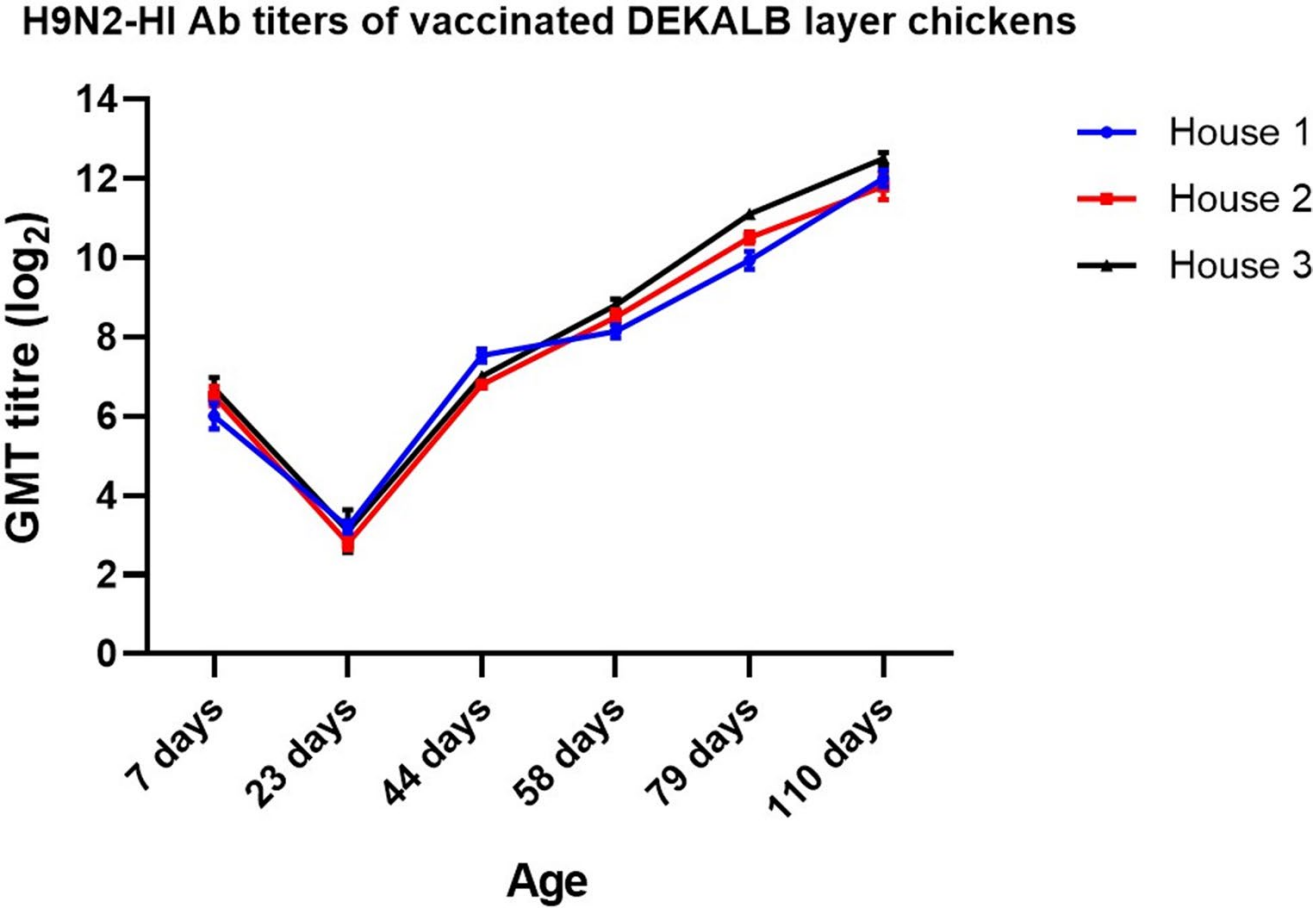
Serology of the commercial layer houses (DEKALB) as determined by the HI test. Serological response of DEKALB layer chickens prime and boost vaccinated with inactivated Avian Influenza strain (AIV), strain A/chicken/Egypt/FAO-S33/2021 (H9N2), measured using HI test to determine the level of specific antibodies against the applied vaccine at different ages. The titres are expressed as GMT ± SE and the data were visualised using GraphPad Prism version 8

To evaluate the efficacy of the PF (H9N2) vaccine in eliciting robust immune responses against the AIV subtype H9N2 in commercial layer chickens, specific AIV-HI titres were measured at different time intervals of the bird life. The vaccination protocol involved two doses administered at 24 and 58 days of age, following the decline of MDA to levels ˂ 4 log_2_. Prior to vaccination, the HI titres of MDA against AIV (H9N2) were recorded at 6.0 ± 0.33 log_2_, 6.5 ± 0.30 log_2_, and 6.7 ± 0.30 log_2_ across the three experimental houses. Following the initial vaccination at 24 days, the geometric mean antibody titres (GMTs) were observed to rise to 7.5 ± 0.20 log_2_, 6.8 ± 0.12 log2, and 7.0 ± 0.12 log_2_ by 40 days of age in the respective houses. At the time of the booster vaccination at 58 days, the GMTs further increased to 8.1 ± 0.17 log_2_, 8.5 ± 0.18 log_2_, and 8.8 ± 0.15 log_2_ for houses 1, 2, and 3, respectively. Eighteen days post-boosting with the PF (H9N2) vaccine, the GMTs exhibited a significant increase, reaching approximately double the previous values at 9.9 ± 0.22 log_2_, 10.5 ± 0.15 log_2_, and 11.1 ± 0.12 log_2_ for houses 1, 2, and 3, respectively. The GMTs continued to rise, reaching 12 ± 0.20 log_2_, 11.8 ± 0.34 log_2_, and 12.5 ± 0.15 log_2_ at 110 days of age for houses 1, 2, and 3, respectively (Fig. 12).

## Discussion

Avian influenza viruses H5N1, H5N8, and H9N2 are among the most prevalent AIV poultry adapted-endemic strains circulating globally (Peacock et al. 2019; Charostad et al. 2023). Their extensive dissemination poses significant economic challenges to the poultry industry and constitutes major risks to public health. The first commercial vaccination applied for the prevention of the H9N2 AIV was in China using three viruses (A/chicken/Guangdong/SS/1994, A/chicken/Shandong/6/1996, and A/chicken/Shanghai/F/98). Since then, numerous inactivated H9N2 avian influenza virus (AIV) vaccines have been developed worldwide, utilizing various antigenic seeds that correspond to the circulating strains. Despite the deployment of these diverse vaccines, which are derived from different sources, the H9N2 virus continues to pose significant threats in the Middle East, resulting in substantial economic repercussions (Li et al. 2020). Previous studies have indicated that over the past few decades, H9N2 has been repeatedly introduced among Saudi Arabia, the United Arab Emirates, and Iraq, which serve as primary distribution centres facilitating the global migration of the virus, characterized by both bi-directional and long-distance spread. Furthermore, it has been reported that H9N2 was introduced to Egypt via Israel in mid-2009, with the descendants of the Egyptian live attenuated influenza vaccines (H9N2) being transmitted back to Israel in 2015 (Li et al. 2020). This information underscores the significance of the vaccine seed origin as a critical concern at both national and global levels. In the experimental studies presented, the maternal-derived antibodies (MDA) were measured at approximately 9 log_2_ against the two antigens utilized. These levels exhibited a regression of 1–2 log at 14 days of age, followed by a rebound at 21–28 days. Notably, high MDA levels against the H9N2 virus have previously been shown to interfere with the immune responses elicited by the inactivated H9N2 vaccine in broiler chickens (Pan et al. 2022). However, the short lifespan of broiler chickens complicates the administration of booster doses. Nonetheless, a single vaccination with the PF (H9N2) vaccine at three different time points has been shown to provide the birds with protective antibody GMTs exceeding 5 log_2_ up to 28 days of age. The previous offering defense against other homologous antigens and genetically similar strains identified in recent years.

Reliance solely on humoral immune responses is insufficient for effective protection against H9N2 infection. Nevertheless, the reduction of clinical symptoms, histopathological damage, and viral shedding in immunized birds upon infection are critical indicators of success. Therefore, a challenge study was performed to evaluate the clinical protection of the studied vaccine against recent AIV H9N2-G1 lineage strain. H9N2 AIV is a low pathogenic AIV that does not cause chicken deaths when infected alone. However, co-infection with other pathogens can cause mortality in chickens (Jaleel et al. 2017). To insure the success of the respiratory infection, the LaSota vaccine was administered in two doses, 48 h prior to the onset of infection, serving as a trigger in the two conducted challenge experiments (Ellakany et al. 2018). Hence, the H9N2 infection in commercial broiler chickens that had been previously vaccinated with LaSota resulted in significant degeneration of the mucous glands and a partial loss of the tracheal mucosal lining. In the absence of H9N2 immunization, AIV (H9N2) early challenge resulted in peak tracheal shedding at 3 and 7 dpi with 100% shedders followed by a reduction of 4 log_10_ in shedding and a decrease to 33% shedders by ten dpi. The early vaccination with inactivated H9N2 vaccine led to early protection against early virus challenge infection with significant tracheal shedding, especially in the early phase of infection. However, the zero and three days old immunization reduced the duration of virus shedding and virus shedding was minimized and even disappeared. This reduction was not existed in the instance of the seven days old vaccination, probably the vaccine was not established its active immunization yet. Taken together, the presented data proofed the efficacy of the early AIV H9N2 vaccination, especially in the instance of the early challenge. The presence of positive individual shedders at 7 days old post vaccination in the differentially vaccinated groups led to transmission of the virus to the contact non-immunised birds. The aforementioned confirmed the lack of ability of the inactivated H9N2 vaccine to induce sterilized immunity even with presence of genetic matching seed strain (Zhang et al. 2024).

When the broilers were exposed to later challenges, 21 days old, and in accordance with the clinical index, the full dose three days old vaccination led to the lowest mean of viral shedding. All the vaccinated groups reduce the duration of virus shedding and number of shedders to 2/6, 7dpi. The providing data suggested that dose of 10^6^ EID_50_ can still infect vaccinated chickens even with HI Ab GMTs ranged from 5 to 6 log_2_. Even though, those vaccinated chicken had limited and shorter shedding time, which could be potentially minimized to 3 dpi. Consequently, the virus transmissibility to contact non-immunised birds was completely inhibited in this experiment. Earlier study performed in China showed that transmission is not sufficiently reduced by the H9N2 vaccine, even when vaccinated chickens have an IgG serum titre > 3 log_2_, which is considered protective for vaccination against homologous HPAI virus. The difference between the two experiment can be regarded to our study doses, on the other hand, cast new light on virus transmission and immune escape of AIV (H9N2) subtype in vaccinated chickens populations, and shows that new mitigation strategies against LPAI viruses in poultry are needed.

Earlier studies showed that the H9N2 AIV induced histopathological alterations predominantly in the respiratory tract, which were characterized by inflammatory and necrotic processes. The proliferation of goblet cells was seen first in the trachea, followed by the sloughing of the tracheal epithelium and deciliation with the lymphocytic infiltration of the mucosa. The lungs usually have congestion and perivascular haemorrhages with heterophil cell infiltration and the collapse of the alveoli (Awadin et al. 2018; Bóna et al. 2023). In the current work, the two challenge studies exhibited similar lesions to the aforementioned. Additionally, the deterioration of tracheal cilia and the presence of fibrino-leucocytic exudate within the tracheal lumen were significantly more pronounced in the challenges conducted at 21 days of age. This evidence suggests that a delayed infection with H9N2 avian influenza virus can lead to greater cytological damage in the respiratory tract of birds.Vaccination with the inactivated PF (H9N2) vaccine using different dosages and timing minimize the histopathological lesion induced by the challenge strain. Birds vaccinated with the full dose (9–10 HAU) at three days old had the lowest necrotic and inflammatory changes in the respiratory tract. These results coordinated with the clinical indices results and virus shedding’s at certain time points.

In order to investigate the vaccine provided protective efficacy after the complete waning of the MDA as well as to investigate the priming-boost vaccination protocol, a field trial on three houses of layer flocks was performed. Single dose of vaccine could not be sufficient for layer chickens entire life span. It has been found earlier infection with AIV (H9N2) and virus shedding may be avoided by early booster administration (Xie et al. 2021). The HI-Ab titres in the three studied houses post priming-boosting vaccination within more than one month in between were ranged from 11.8 to 12.5 log_2_. The boosting vaccination enhanced the HI-Ab titres to more than 4log, which were a very good sufficient as a prelaying protective titter. This field trials reconfirmed the role of the MDA in the hindrance if the humoral immune response induced by the inactivated vaccine. Hence, injection of the vaccine at 3 days old after the complete waning of the passive immune responses led to boosting of the HI-Ab up to 4 log at 21 days old. This observation was not existed in the instance of MDA existence.

Taken together, we have demonstrated that early vaccination of broiler chickens with an inactivated H9N2 vaccine, using a homologous virus seed that matches the circulating strain, can induce protective humoral immune responses even in the presence of maternal antibody interference. The applied vaccine effectively reduced virus shedding and transmissibility to contact birds, as well as minimized gross and histopathological lesions following viral challenge. A prime-boost vaccination strategy for layers could elicit sufficient humoral immune responses to achieve protective pre-laying antibody titres. Continuous updating of the AIV vaccine seed is necessary to maintain effective protection against AIV (H9N2) challenges. The absence of maternal immunity, combined with the prime-boost vaccination approach, may further enhance immune responses.

## Data Availability

The data that support the findings of this study are available on request from the corresponding author [O.H.].
